# SUMOylation of Dorsal attenuates Toll/NF-κB signaling

**DOI:** 10.1093/genetics/iyac081

**Published:** 2022-05-14

**Authors:** Sushmitha Hegde, Ashley Sreejan, Chetan J Gadgil, Girish S Ratnaparkhi

**Affiliations:** Biology, Indian Institute of Science Education & Research, Pune 411008, India; Chemical Engineering and Process Development Division, CSIR—National Chemical Laboratory, Pune 411008, India; Chemical Engineering and Process Development Division, CSIR—National Chemical Laboratory, Pune 411008, India; CSIR—Institute of Genomics and Integrative Biology, New Delhi 110020, India; Biology, Indian Institute of Science Education & Research, Pune 411008, India

**Keywords:** Drosophila, haploinsufficiency, innate immunity, SUMO, transcription

## Abstract

In *Drosophila*, Toll/NF-κB signaling plays key roles in both animal development and in host defense. The activation, intensity, and kinetics of Toll signaling are regulated by posttranslational modifications such as phosphorylation, SUMOylation, or ubiquitination that target multiple proteins in the Toll/NF-κB cascade. Here, we have generated a CRISPR-Cas9 edited Dorsal (DL) variant that is SUMO conjugation resistant. Intriguingly, embryos laid by *dl^SCR^* mothers overcome *dl* haploinsufficiency and complete the developmental program. This ability appears to be a result of higher transcriptional activation by DL^SCR^. In contrast, SUMOylation dampens DL transcriptional activation, ultimately conferring robustness to the dorso-ventral program. In the larval immune response, *dl^SCR^* animals show an increase in crystal cell numbers, stronger activation of humoral defense genes, and high *cactus* levels. A mathematical model that evaluates the contribution of the small fraction of SUMOylated DL (1–5%) suggests that it acts to block transcriptional activation, which is driven primarily by DL that is not SUMO conjugated. Our findings define SUMO conjugation as an important regulator of the Toll signaling cascade, in both development and host defense. Our results broadly suggest that SUMO attenuates DL at the level of transcriptional activation. Furthermore, we hypothesize that SUMO conjugation of DL may be part of a Ubc9-dependent mechanism that restrains Toll/NF-κB signaling.

## Introduction

Toll-like receptor signaling is a highly conserved, ancient response to combat pathogenic attacks in multicellular eukaryotes (Kopp and Ghosh 1995; Medzhitov *et al.* 1997; Zhang and Ghosh 2001; Janeway and Medzhitov 2002). In *Drosophila*, in addition to its role in regulating the host response to infection, the Toll/Dorsal pathway has been co-opted to orchestrate early development, laying down the foundations for the dorso-ventral (DV) body plan *(*reviewed by Steward and Govind 1993; Morisato and Anderson 1995; Belvin and Anderson 1996; Rusch and Levine 1996; Stathopoulos and Levine 2002; Valanne *et al.* 2011). The chief effector in DV development, the NF-κB transcription factor (TF) Dorsal (DL) is held inactive in the cytoplasm by the IκB ortholog Cactus (Cact) (Steward 1987; Rushlow *et al.* 1989; Roth *et al.* 1989, 1991; Geisler *et al.* 1992; Govind *et al.* 1993; Whalen and Steward 1993). The asymmetric binding of the ligand Spaetzle to the Toll receptor sets in motion a kinase cascade, leading to the formation of a DV gradient of DL (Steward *et al.* 1988; Roth *et al.* 1989; Bergmann *et al.* 1996) in the syncytial blastoderm, where DL activates 50–70 target genes to specify the presumptive germ layers of the fly (Kosman *et al.* 1991; Ip *et al.* 1992; Araujo and Bier 2000).

The Toll signaling arm is also deployed later in the *Drosophila* life-cycle to ward off fungal and Gram-positive bacterial insults, triggering the humoral immune response (Lemaitre *et al.* 1996; Rutschmann *et al.* 2002; Ferrandon *et al.* 2007). Here, DL acts in concert with DL-related Immunity Factor (Dif) to aid in host defense (Lemaitre *et al.* 1995; Anderson 2000). Toll/NF-κB signaling is subject to regulation by posttranslational modifiers (PTMs) (Karin and Ben-Neriah 2000; Zhou *et al.* 2005). The best characterized example is the phosphorylation of Cact in response to activation of the receptor, which leads to degradation of Cact and release of DL for its journey to the nucleus (Belvin *et al.* 1995; Liu *et al.* 1997).

SUMO resembles ubiquitin in its 3-dimensional structure but utilizes a related but distinct set of conjugating enzymes to attach to target proteins at a lysine residue, most often part of a ψ-K-X-E/D consensus motif (Johnson 2004; Hay 2005; Varej**ã**o *et al.* 2020). The covalent conjugation of SUMO to targets confers novel properties, in terms of altered localization, stability, or activity. The modified protein can further be recognized by a cognate partner via a SUMO-interaction motif, thus changing its interaction potential (Gareau and Lima 2010). SUMO regulates a plethora of cellular processes and is upregulated in response to protein-damaging stresses like heat shock, osmotic stress, proteasomal inhibition, and immune stress (Enserink 2015; Hegde *et al.* 2020). This increase in global SUMOylation, termed the SUMO-stress response, is an essential cyto-protective adaptation (Ryu *et al.* 2020).

DL has been shown to be a SUMO target, based on experiments conducted in *Drosophila* S2 cells (Bhaskar *et al.* 2000, 2002). Studies in larvae have also emphasized the interplay of SUMO and the Toll pathway in modulating host defense. Mutations in the SUMO E2 ligase Ubc9, encoded by *lesswright (lwr)* in *Drosophila*, lead to the over-proliferation of hemocytes. Introducing mutations in the *dl* and *Dif* loci in a *lwr* mutant background restores the wild-type blood cell population, providing evidence for the intersection of Toll signaling with the SUMO conjugation machinery (Huang *et al.* 2005; Chiu *et al.* 2005). In the embryo, mass spectrometric studies suggest that DL is SUMO conjugated (Nie *et al.* 2009). However, roles for DL SUMOylation in the animal have not been studied.

Here, we sought to delineate the function of SUMO conjugation of DL in *Drosophila*. In our study, we employ a CRISPR-Cas9-based strategy, which allows precise editing of the DL locus, replacing the 382^nd^ lysine, the site for SUMOylation, with a charge-preserving arginine. This *dl^K382R^* animal is then subsequently evaluated for its effect on early development and host defense, both of which represent critical spatiotemporal domains for Toll/DL signaling. Our studies uncover roles for SUMO conjugation of DL in supporting the robustness of embryonic DV patterning. Furthermore, we find that DL SUMOylation has roles in both the cellular and humoral response in the larvae. In these 2 distinct signaling contexts, a common mechanism that emerges is the role of DL SUMO conjugation in negatively regulating Toll signaling by specifically attenuating DL mediated transcriptional activation.

## Materials and methods

### Fly husbandry and stocks

Flies were raised on standard cornmeal agar at 25°C unless stated otherwise. For the *dl* deficiency experiments, flies were crossed and maintained at 29°C. The following fly stocks were procured from the Bloomington *Drosophila* Stock Centre: *dl^1^/CyO* (3236), *dl^4^/CyO* (7096), and *vasa-Cas9* (51323). The *dl* deficiency allele *w-, y; J4/CyO* containing a precise deletion of the *dl* and *dif* loci, was a kind gift from the Govind laboratory, City University of New York (CUNY), NY.

### Generation of transgenic CRISPR lines

The Fly CRISPR Optimal Target Finder was used to design the gRNA with zero predicted off-target effects. The gRNA sequence 5_**′**_-GAAACATACCGCCCATTAAAA-3_**′**_ was incorporated into the forward primer sequence GAAACATACCGCCCATTAAAAGTTTTAGAGCTAGAAATAGC. A reverse primer of the following sequence was used: GAAGTATTGAGGAAAACATA. The gRNA was cloned into the pBFv-U6.2 vector as described previously (Kondo and Ueda 2013), using the primers listed above. The 100-mer ssODN sequence is as follows: TTTAACTAGGTTTTTTTTTTGTAGTTTTAGTGTATAAAACTCACCTCTTGGTTCCGTTCGAATGGGCGGTATGTTTTGTGTATTCCAGCAATTCATGTT*A*.

A total of 620 *vasa-Cas9* embryos were co-injected with the gRNA and ssODN, at the C-CAMP facility, NCBS. A total of 450 F0 adults that emerged were crossed with *Tft/CyO* balancer flies individually. Three emergent flies from each cross were balanced further, with the *Tft/CyO* balancer, and maintained as separate lines. Homozygous flies from these founder lines were screened for the presence of the mutation by PCR followed by restriction digestion. For the isolation of genomic DNA, flies were placed in 0.2-mL tubes individually and lysed in 50 μL of squishing buffer (10 mM Tris-Cl pH 8, 1 mM EDTA, 25 mM NaCl, and 0.2 mg/mL Proteinase K). After incubation at 37°C for 30 min, Proteinase K was inactivated by heating at 85°C. One microliter of the genomic DNA was used in a 10_**-**_μL PCR. The following primers were used for the PCR: F: CAGTTCTGAGTAAGTCTTTATCGGAGTTCA; R: CCAAAGGGTTGTGGCGAGGTAT. The PCR product was digested with the restriction enzyme BstBI and resolved on a 1.2% agarose gel. Four transformants were obtained after screening 200 lines.

### Cuticle preparation

Embryos were collected for 3 hr and aged for 22 hr at 25 or 29_** °**_C, depending on the nature of the experiment. They were dechorionated in a 4% sodium hypochlorite solution for 2 min. Dechorionated embryos were washed thoroughly under running tap water and transferred to a scintillation vial containing 1:1 methanol: heptane. The vial was shaken vigorously for a few minutes, and de-vitellinized embryos in the lower methanol phase were transferred to a new vial with fresh methanol. Embryos were transferred onto a slide, mounted in 85% lactic acid, and incubated overnight at 55°C on a slide warmer. Cuticles were imaged on a Zeiss Axio Imager Z1 microscope, using dark field illumination, with a 10× objective.

### Embryo staining

The 0–3-hr embryos were dechorionated in 4% sodium hypochlorite for 2 min. Embryos were rinsed and fixed in a 1:1 solution of 4% formaldehyde in 1× phosphate-buffered saline (PBS):heptane for 20 minutes. The aqueous phase containing formaldehyde was removed, and embryos were devitellinized by adding an equal volume of ice-cold methanol followed by vigorous shaking. Devitellinized embryos were washed thrice in methanol. Embryos were re-hydrated and permeabilized by giving six 15-min washes in 1× PBS containing 0.3% Triton X-100 (0.3% PBS-T). After blocking with 2% bovine serum albumin (BSA) in 0.3% PBS-T, embryos were incubated overnight at 4°C with the primary antibody. Following four 15-min washes with 0.3% PBS-T, embryos were incubated with the secondary antibody for an hour at room temperature. Embryos were washed thrice in 0.3% PBS-T, and DAPI was added in the penultimate wash. Embryos were mounted in SlowFade Gold mountant (Invitrogen) and imaged on a Leica Sp8 confocal microscope under a 20× oil-immersion objective. To obtain transverse cross sections, embryos were sectioned with a razor as previously described (Liberman *et al.* 2009; Reeves *et al.* 2012; Trisnadi *et al.* 2013) and mounted in 70% glycerol. Embryos were imaged on a Leica Sp8 confocal microscope with a 40× oil-immersion objective. The following antibodies were used: Mouse anti-Dorsal, 1:1,000 (DSHB 7A4-c) and goat antimouse Alexa568 secondary antibody, 1:1,000 (Invitrogen).

### Image analysis of fixed embryos

Images of transverse sections were analyzed as described in Trisnadi *et al.* (2013), with minor modifications. Briefly, the StarDist plugin in ImageJ was used to obtain nuclear masks of the DAPI channel with distinct numerical labels. For each nucleus identified, corresponding DL fluorescence intensity values were obtained. Normalized values of DL nuclear intensities were calculated as a ratio of DL intensity to that of the nuclear channel. The DL gradient was fit to a Gaussian, using GraphPad Prism8, to obtain the amplitude and width parameters. The amplitude is the height of the curve’s peak, while σ is the measure of 1 standard deviation, determining the width of the distribution.

### RNA in situ hybridization

Embryos were collected and aged at 29°C. Antisense digoxigenin-labeled RNA probes for *twi*, *sna*, *sog*, and *zen* were used and hybridization was carried out as previously described (Tautz and Pfeifle 1989). Antidigoxigenin-alkaline phosphatase antibody (Merck) was used at a concentration of 1:2,000 and NBT/BCIP (Merck) was used as the color-development substrate for AP. Images were acquired on a Zeiss Axio Imager Z1 microscope, using DIC optics, with a 10× objective.

### Western blots and their analysis

Fat bodies (8–10 per sample) were dissected in ice-cold PBS and crushed in lysis buffer (2% SDS, 60 mM Tris-Cl, pH 6.8, and 1× PIC). Samples were cleared by centrifuging at 21,000 *g* for 30 min. Total protein was estimated by BCA assay (Pierce) and samples were boiled in 1× Laemmli buffer. Equal amounts of protein (30–40 μg/sample) were resolved on a 10% polyacrylamide gel and transferred onto a PVDF membrane (Immobilon-E, Merck). The membrane was blocked with 5% milk in TBS containing 0.1% Tween20 (TBS-T) for an hour followed by incubation with the primary antibody diluted in 5% milk in TBS-T. Following 3 washes with TBS-T, the membrane was incubated with the secondary antibody diluted in 5% milk in TBS-T for an hour, at room temperature. The membrane was washed thrice with 0.1% TBS-T, incubated with Immobilon Western Chemiluminescent HRP substrate (Merck), and visualized on a LAS4000 Fuji imaging system. The following antibodies were used: Rabbit anti-Dorsal, 1:5,000 (kind gift from the Courey laboratory); Mouse anti-Cactus, 1:100 (DSHB 3H12); Mouse anti-α-Tubulin, 1:10,000 (T6074, Sigma-Aldrich); Goat antirabbit HRP; and Goat antimouse HRP secondary antibodies, each at 1:10,000 (Jackson ImmunoResearch).

### Microbial infection

*Staphylococcus saprophyticus* (ATCC 15305) was used for the septic injury experiments. For larval infection, the bacteria were grown overnight, concentrated by centrifugation, and the pellet washed with PBS. Larvae were placed on a cold agar plate and infected at the posterior region with a fine insect pin dipped in the concentrated culture, as described previously (Kenmoku *et al.* 2017). Infected larvae were transferred to a fresh sugar-agar plate, at 25°C and processed at the appropriate time points.

### Fat body staining

Fat bodies from wandering third instar larvae were dissected in ice-cold PBS and fixed in 4% formaldehyde in PBS, for 20 min. The tissue was permeabilized by washing thrice in 0.1% PBS-T followed by blocking in 2% BSA in 0.1% PBS-T. The tissue was incubated overnight with the primary antibody diluted in 2% BSA in 0.1% PBS-T, at 4°C. Following three 15-min washes with 0.1% PBS-T, secondary antibody diluted in 2% BSA in 0.1% PBS-T was added and incubated for an hour at RT. After three 15-minute washes with 0.1% PBS-T, with DAPI being added in the second wash, the tissue was mounted in SlowFade Gold mountant (Invitrogen) and imaged on a Leica Sp8 confocal microscope under a 20× oil-immersion objective. The antibodies used were: Mouse anti-Dorsal, 1:1,000 (DSHB 7A4-c) and goat antimouse Alexa488 secondary antibody, 1:1,000 (Invitrogen). Mean pixel intensity for DL staining in the cytoplasm and the nucleus was quantified using ImageJ software. The cytoplasmic intensity was averaged across 3 circular ROIs per cell and the same ROI was used to calculate the nuclear intensity. Five to seven cells per fat body were analyzed for at least 7–9 fat bodies across 3 biological replicates.

### Quantitative PCR

RNA was extracted from appropriately staged embryos or whole larvae (*n* = 10/sample) using the RNeasy Plus Universal mini kit (Qiagen) according to the manufacturer’s instructions. 1 μg of total RNA was used to generate cDNA using the High-Capacity cDNA Reverse Transcription kit (Thermo Fisher Scientific). The qPCR reaction was performed on a qTOWER^3^ real-time thermal cycler (Analytik Jena) with KAPA SYBR FAST master mix (Sigma-Aldrich). Gene expression was monitored using gene-specific primers. Transcript levels were calculated using the comparative Ct method to obtain fold change values. Relative mRNA levels were calculated using the delta Ct values. Rp49 was used as a reference gene. The following primer pairs were used (Forward primer, F and reverse primer, R):

*rp49* F: GACGCTTCAAGGGACAGTATC, *rp49* R: AAACGCGGTTCTGCATGAG;

*dl* F*: ATCCGTGTGGATCCGTTTAA, dl* R: AATCGCACCGAATTCAGATC;

*twi* F: AAGTCCCTGCAGCAGATCAT, *twi* R: CGGCACAGGAAGTCAATGTA;

*sna* F: CGGAACCGAAACGTGACTAT, *sna* R: CCTTTCCGGTGTTTTTGAAA;

*zen* F: TACTATCCAGTTCACCAGGCTAA, *zen* R: TCTGATTGTAGTTGGGAGGCA;

*mtk* F: GCTACATCAGTGCTGGCAGA, *mtk* R: TTAGGATTGAAGGGCGACGG;

*drs* F: CTGTCCGGAAGATACAAGGG, *drs* R: TCGCACCAGCACTTCAGACT.

### Quantitative RNA sequencing and analysis

Cages containing flies of the appropriate genotype were set up with sugar-agar plates. Plates were changed twice after 1-hr intervals and the third collection was used for the experiment. Embryos were collected at 29°C for 2 hr. RNA was isolated from 2 biological replicates for each sample using the RNeasy Plus Universal mini kit (Qiagen). RNA concentration was determined using a NanoDrop instrument, and 500 ng of RNA was used to generate the cDNA library with the QuantSeq 3_**′**_mRNA-Seq Library Prep Kit FWD for Illumina (Lexogen), according to the manufacturer’s instructions. The library size and quality were determined on a Bioanalyzer with a high sensitivity chip (Agilent) and concentration assessed using a Qubit fluorometer, with a dsDNA High Sensitivity assay kit (Thermo Fisher Scientific). The equimolar, pooled library was sequenced on an Illumina NextSeq 550 system, generating 75 bp single-end reads. The sequencing files obtained were uploaded onto BlueBee’s genomics analysis platform (https://lexogen.bluebee.com/quantseq/). Reads were trimmed in BlueBee using bbduk (v35.92). Reads were aligned, counted, and mapped using BlueBee’s STAR-aligner (v2.5.2a), HTSeq-count (v0.6.0), and RSEQC (v2.6.4), respectively. A DESeq2 application within BlueBee (Lexogen Quantseq DE 1.2) was used to obtain normalized gene counts and identify differentially expressed genes (DEGs) based on a false discovery rate (FDR) cutoff *P-*adjusted value <0.1. Downstream analysis was performed on EdgeR. Raw count data were transformed using the logCPM function to obtain values for the heatmap, generated using pheatmap in RStudio. GO enrichment analysis was performed using gProfiler (https://biit.cs.ut.ee/gprofiler/gost).

### Blood cell preparation and counting

Third instar larvae were cleaned with copious amounts of water and a brush, and placed individually in a drop of 20 μL ice-cold PBS (5 per replicate) on a clean glass slide. Larvae were carefully ripped open in PBS using watchmaker’s forceps, without damaging the internal organs. The carcass was discarded, and 10 μL of the PBS solution containing blood cells was transferred to a Neubauer hemocytometer chamber (Hausser Scientific). Plasmatocytes were counted on a Zeiss Axio Vert.A1 microscope at 40× magnification using phase-contrast optics. To visualize crystal cells, wandering third instar larvae (8 per replicate) were heated at 60°C in a water bath for 10 min. Images were acquired on a Zeiss Axio Vert.A1 microscope at 10× magnification. Crystal cells in 3 terminal segments were counted and plotted.

### Statistical analysis

All experiments were performed in 3 biological replicates, unless stated otherwise. Data are presented as mean ± SEM. Statistical analysis was performed using GraphPad Prism8.

### Mathematical modeling and simulation

Mathematical models for DL (or NF-κB) signaling (Schloop *et al.* 2020) have earlier been used to study the intracellular signaling kinetics of this pathway. Our objective was to simulate the effect of SUMOylation, and compare the response (reporter expression) of DL^WT^ and DL^SCR^. To this end, we developed a simplified model (Fig. 8a, see Supplementary SI-1 for all reactions) as described below. DL can exist either as monomers, homo-dimers of unSUMOylated DL (DL^U^: DL^U^) or SUMOylated DL (DL^S^: DL^S^) or as a DL^U^: DL^S^ heterodimer. The rates and therefore the equilibrium constant of the dimerization reactions may be different for DL^U^ and DL^S^ monomers. The equilibrium constants for these reactions are denoted by $K_{D}^{u}$, $K_{D}^{s}$, and $K_{D}^{us}$ with superscripts indicating the nature of the monomers. Single u and s are used to denote the homodimer forms. Other processes included in the model are the dimers binding to Cact **(**equilibrium constants $K_{i}^{u}$, $K_{i}^{s}$. or $K_{i}^{us}$ depending on the dimer**)**, dimers partitioning to the nucleus **(**with partition coefficients $K_{t}^{u},\;K_{t}^{s}$, and $K_{t}^{us}$ for the 3 dimer types**)**, dimers in the nucleus binding to the promoter site P **(**with equilibrium constants $K_{p}^{u}$, $K_{p}^{s}$, and $K_{p}^{us}$) and reporter expression at rates $k^{u}$, $k^{s}$, and $k^{us}$ corresponding to the dimer bound to the promoters.

These parameters were estimated from reported values for the same proteins (Supplementary SI-2), with values for mammalian systems used whenever necessary. We assume that the equilibrium constants for reactions involving DL^S^ homodimers and heterodimers are the same, but may be different from the equilibrium constant for the corresponding reaction where the DL^U^ homodimer is a reactant or product. Thus, $K_{p}^{u}\neq K_{p}^{us}=\;K_{p}^{s}$, $k^{u}\neq k^{us}=k^{s}$ and so on. Parameters for the unSUMOylated DL reactions were based on previous reports (Supplementary SI-2), and values for the SUMOylated DL reactions were explored in hundred-fold range relative to this value.

The change in the concentration of individual forms of DL (i.e. nuclear and cytoplasmic dimers, cactus bound and promoter bound), Cact, and the promoter is given by the difference in the rate at which other forms convert to that particular one, and the rate at which it is converted to another form. Assuming mass action kinetics for all reactions, this mass balance on individual forms can be mathematically expressed as a set of coupled differential equations [Supplementary Equations (1)–(18)]. For instance, the rate of change of DL^U^ in the cytoplasm is the difference in the rates at which it is formed due to dimer dissociation [second and fourth terms in Supplementary Equation (1)] and the rates at which it is converted to dimers (first and third terms). Using the steady state assumption, the net rate is set to zero. Similar balances are written for other forms. Four equations [Supplementary Equations (19)–(22)] represented conservation of total DL^U^, DL^S^, Cact, and promoter sites. These equations can be simplified by substitution. For instance, rearrangement of the terms in Supplementary Equation (3) leads to an expression for DL^U^ homodimer in terms of the equilibrium constants and concentrations of DL^U^ monomer, nuclear homodimer and bound Cact. After many such successive substitutions, we get 2 equations [Supplementary Equations (23) and (24)] in 2 unknown concentrations, which can be numerically solved numerically using the fsolve function in MATLAB 2020b. Since this is a (pseudo) steady state model, it is unable to simulate dynamic changes in the concentrations. Numerical solution of Supplementary Equations (23) and (24) give the steady state cytoplasmic monomer concentrations. These can be substituted to obtained steady state values for all species. In particular, the concentrations of promoter sites bound to DL^U^ homodimers and dimers containing DL^S^, and thence the reporter expression levels, can be calculated. For WT, it is assumed that total DL comprises 5% DL^S^ and 95% DL^U^. This assumed percentage is varied and results recalculated to check dependence of qualitative results on this assumption. In the SCR mutants, DL^S^ is absent. Total DL^S^ is set to zero, and the steady state reporter expression is calculated keeping all other parameters and total concentrations unchanged. The ratio **(**$R^{\mathrm{SCR}}/R^{WT}$**)** of the steady state reporter expression for SCR and WT is represented on the *y*-axis of Fig. 8b and all simulation results (Supplementary Figs. 7 and 9–13).

## Results

### Generation of a genome-edited *dl^K382R^* mutant

**Fig. 1.**
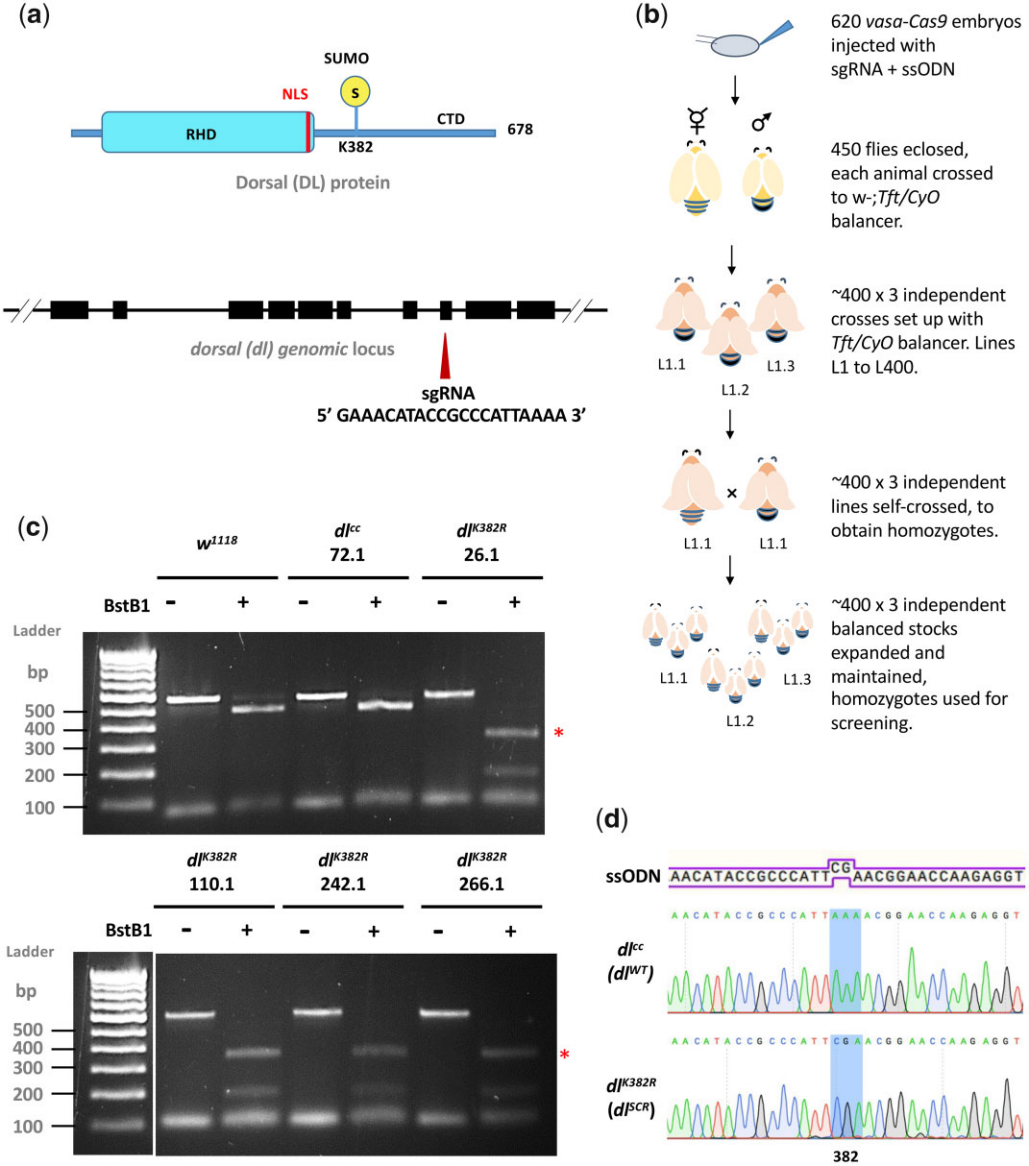
Creating the *dl^K382R^* mutant using the CRISPR-Cas9 system. A schematic representation of the DL protein (in blue) and gene locus (in black) is presented in (a). DL is SUMOylated at K382, part of the consensus motif I**K**TE. A 20-bp *sgRNA* was designed to create a double-strand break in the vicinity of *dl^K382^*, in exon 8. The detailed crossing scheme for the generation of the *dl^K382R^* allele after injection of the gRNA plasmid and ssODN is outlined in (b). Homozygous flies obtained were screened by genomic PCR and digestion with the BstBI enzyme, which recognizes the engineered site of mutation, TTCGAA (c). Four independent lines—26.1, 110.1, 242.1, and 266.1 showed a distinct digest of the PCR product (indicated by red asterisks), while line 72.1 served as a control. d) The presence of the mutation was confirmed through sequencing (codon CGA is highlighted).

In recent years, the advent of CRISPR-Cas9 genome editing technology has allowed the generation of point mutations in a straightforward and site-directed manner (Bassett *et al.* 2014; Gratz *et al.* 2014; Bier *et al.* 2018). DL is SUMOylated (Bhaskar *et al.* 2000; Smith *et al.* 2004) and has a single, well-characterized, and validated SUMO conjugation site at K382 (Fig. 1a) (Bhaskar *et al.* 2002; Anjum *et al.* 2013), supported by SUMO prediction algorithms (Ren *et al.* 2009; Zhao *et al.* 2014; Beauclair *et al.* 2015) as a direct consensus SC-SUMO site. Furthermore, mass spectrometry experiments indicate that DL is SUMOylated in S2 cells (Pirone *et al.* 2017) and in the early embryo (Nie *et al.* 2009). Proteome-wide acetylation studies (Weinert *et al.* 2011) and *Drosophila* PTM databases (Hu *et al.* 2019) do not suggest DL to be acetylated or methylated. Therefore, the DL^K382R^ mutation exclusively abolishes SUMO conjugation, generating a SUMO-conjugation-resistant (SCR) variant of DL and is therefore an ideal target for a CRISPR based mutagenesis experiment. We employed the following genome editing protocol (Fig 1, a and b; *Materials and Methods*) to generate the *dl^K382R^* mutation (Fig. 1, c and d). A single guide RNA (sgRNA) targeting the *dl* locus, with no predicted off-target cleavage sites was cloned into the pBFv-U6.2 plasmid. A 100-bp-long ssODN (Fig. 1a) harboring the K382R mutation was supplied as the repair template and co-injected along with the sgRNA plasmid in embryos expressing Cas9 in the *vasa* domain. The 450 flies (F0) that emerged from the injected embryos were crossed to a second chromosome *w*^-^; *Tft/CyO* balancer (Fig. 1b). Three animals from each vial, for each of the 400 lines, were crossed again to *w*^-^; *Tft/CyO*, to generate stable, putative, *dl^K382R^/CyO* lines. Of these, 200 homozygous, putative transformants were screened for insertion of the ssODN by PCR amplification of the genomic locus followed by restriction digestion by BstBI (Fig. 1c). The screening strategy incorporated a BstBI site in the ssODN, validating the successful incorporation of the mutation in the genome. Based on restriction digestion patterns, ∼2% of lines (4 out of 200), harbored the mutation and we validated these (26.1, 110.1, 242.1, 266.1) by sequencing (Fig. 1c). Representative sequencing data are shown in Fig. 1d. A few lines containing wild-type sequences were also retained and one of these (72.1) was defined as a “CRISPR-control,” *dl^WT^*, at par with the wild-type animal. The *dl^K382R^* genome-edited lines were also used in a trans-allelic combination (e.g. 26.1/110.1) to negate off-target effects. Here onwards, *dl^K382R^* is referred to as *dl^SCR^*, a line where DL is resistant to SUMO conjugation. All *dl^K382R^* and *dl^WT^* lines were homozygous viable with comparable *dl* transcript levels (Supplementary Fig. 1) across developmental stages.

### Early development proceeds normally in *dl^SCR^* embryos

**Fig. 2.**
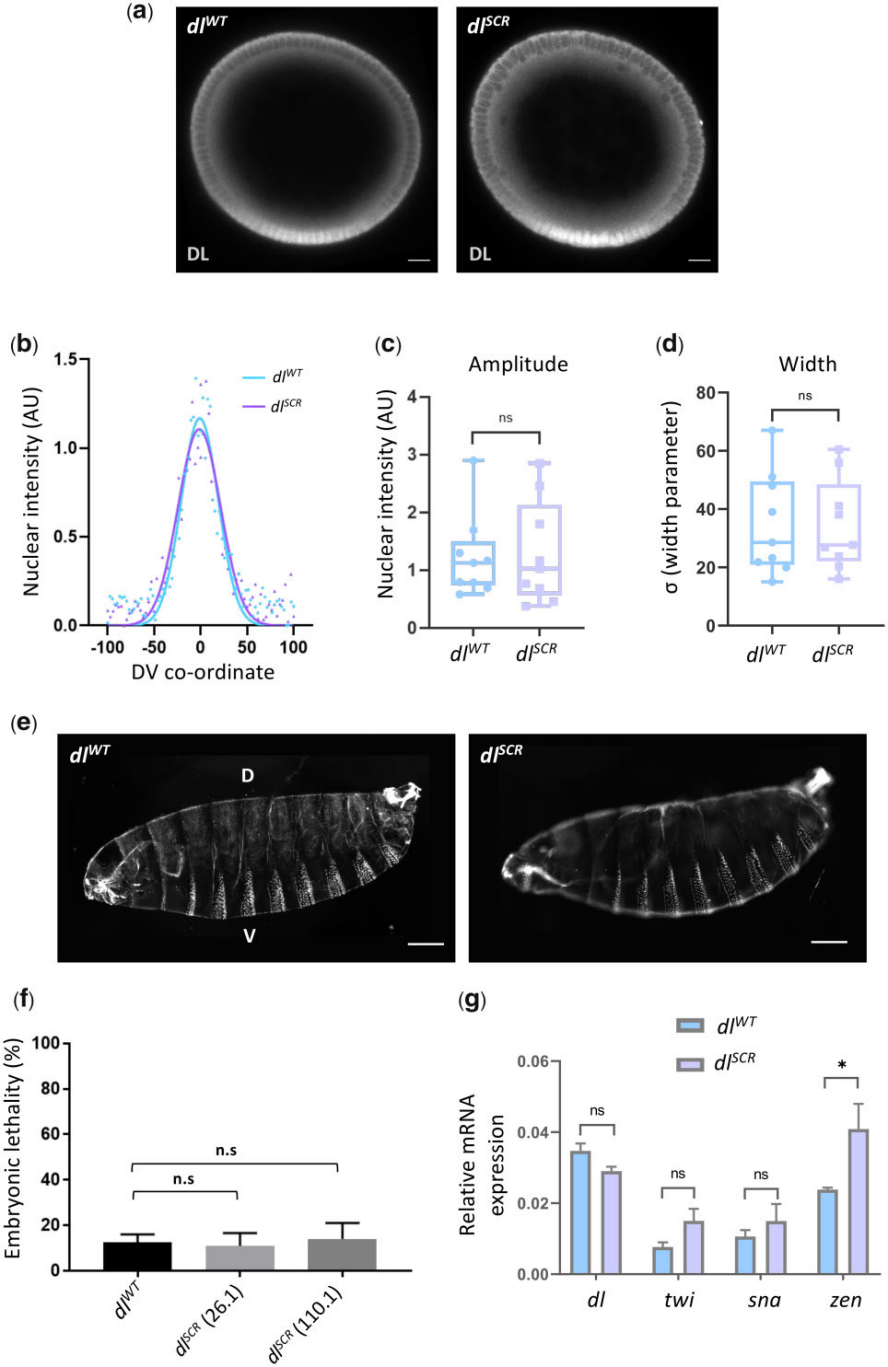
SUMO conjugation is dispensable for embryonic development. Transverse sections of representative cellular blastoderm embryos stained for DL (a). Localization in the nuclei was observed in embryos oriented dorsal-side up and ventral-side at the bottom. b) Representative nuclear intensity profiles of *dl^WT^* and *dl^SCR^* embryos, fitted to a Gaussian. The amplitude (c) and width (d) of the gradient centered at the ventral midline are plotted. *n* = 9, Student’s *t*-test, (ns) *P > *0.05. Cuticle preparations (e) indicate regular arrangement of denticle bands and normal DV patterning. The percentage of unhatched embryos is plotted as embryonic lethality for control and 2 of the mutant lines, 26.1 and 110.1 (f). Genotype of mated mothers is listed on the *X*-axis. *N* = 3, ordinary 1-way ANOVA, (ns) *P > *0.05. g) qRT-PCR analysis of *dl* transcripts and DL target genes *twi*, *sna*, and *zen* for embryos from mated females of the genotypes *dl^WT^* and *dl^SCR^*. *N* = 3, 2-way ANOVA, (ns) *P > *0.05, (*) *P < *0.05.

*dl* is deposited maternally and DL functions as a master regulator in specifying the DV axis (Santamaria and Nüsslein-Volhard 1983; Anderson and Nüsslein-Volhard 1984; Roth *et al.* 1989; Steward and Govind 1993; Morisato and Anderson 1995; Rusch and Levine 1996). Using mass-spectrometry, DL is also among the ∼140 maternal proteins identified as substrates for SUMO conjugation in the 0–3-hr embryo (Nie *et al.* 2009). In eggs laid by homozygous *dl^SCR^* mothers, antibody staining indicates that the DL gradient (Liberman *et al.* 2009; Reeves *et al.* 2012; Trisnadi *et al.* 2013), which could be influenced by SUMO conjugation, appears to be normal (Fig. 2a). A quantitative comparison in embryonic cross-sections (Fig. 2, b–d) confirmed equivalent gradients for DL^WT^ and DL^SCR^. The equivalence of gradients is further supported by the observation that there are no discernible differences in embryonic viability (Fig. 2f andSupplementary Fig. 2a). In addition, the cuticular pattern, a sensitive readout for aberrations in both maternal and zygotic stages (Fig. 2e) is normal for both genotypes. These observations suggest that lack of SUMO conjugation of DL does not significantly change the DV program. Transcript levels, measured by real-time PCR of *dl* and its primary ventral targets *twist (twi)* and *snail (sna)* were similar to controls, while *zerknullt* (*zen)* levels were ∼2-fold higher in *dl^SCR^* mutants (Fig. 2g). Taken together, these results suggest that DL SUMOylation is either dispensable or that the effect of the *dl^SCR^* mutation is compensated for by unknown mechanisms in the developing embryo.

### Haploinsufficiency of *dl* is rescued in *dl^SCR^* embryos

**Fig. 3.**
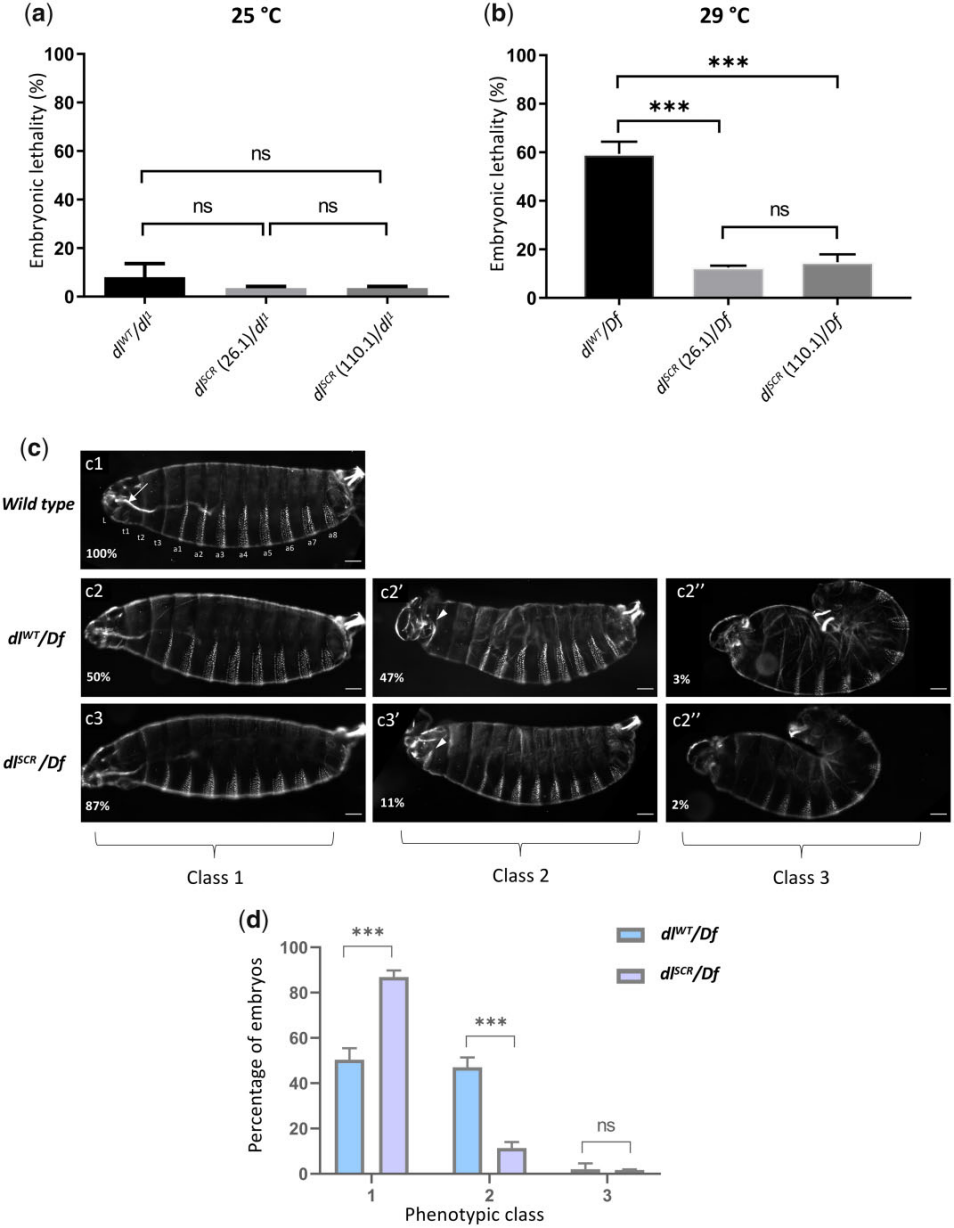
*dl^SCR^ is* haplo-sufficient. Progeny of mothers of the indicated genotype, with 1 functional copy of DL, were scored for viability, 48 hr after egg lay, at 25°C (a) and 29°C (b). *N* = 3, mean ± SEM, ordinary 1-way ANOVA, (ns) *P > *0.05, (***) *P < *0.001. Cuticle preparations of progeny of the maternal genotypes *dl^WT^/Df* and *dl^SCR^/Df*, visualized under a dark-field microscope yielded 3 major ranges of phenotypes, classified as class 1 (intact head region/mouth hook; ventral denticle bands and filzkorper normal), class 2 (defective head structure; denticle bands and filzkorper intact), and class 3 (twisted embryos; defective head structures and filzkorper) (c). Cuticles are oriented dorsal-side up and anterior-side on the left. >100 embryos were scored in each replicate, and the percentage of each phenotypic class is plotted for *dl^WT^/Df* and and *dl^SCR^/Df* (d). *N* = 3, mean ± SEM, 2-way ANOVA, (ns) *P > *0.05, (***) *P < *0.001.

Since SUMO is essential to adapt to a multitude of cellular stresses, we reasoned that a requirement for SUMOylation of DL would only be apparent under conditions of stress (Temp**é** *et al.* 2008). A well-known allelic combination that disrupts the DV developmental program is *dl* haploinsufficiency at 29°C (Nüsslein-Volhard 1979; Nüsslein-Volhard *et al.* 1980; Simpson 1983). Unlike at 25°C, where ∼95% of embryos hatch into larvae (Fig. 3a), at 29°C, ∼50% of embryos laid by *dl^WT^/Df* or *dl^WT^/dl^1^* females fail to hatch (Fig. 3b andSupplementary Fig. 2b). The J4 allele, a deletion spanning *dl*, *dif*, and an uncharacterized transcriptional unit *C2* (Meng *et al.* 1999) was used as a deficiency allele (Df), while *dl^1^* is a null allele. Surprisingly, the embryonic lethality of *dl^SCR^/Df* (Fig. 3b) or *dl^SCR^/dl^1^* (Supplementary Fig. 2b) was significantly lower, at 15% in comparison to the 55% lethality of *dl^WT^/Df* embryos. This result was consistent across 2 of the *dl^SCR^* lines tested, 26.1 and 110.1. At 25°C, the embryonic lethality for both *dl^SCR^/dl^1^* and *dl^WT^/dl^1^* were comparable (Fig. 3a), indicating that a reduction of *dl* gene dosage at 25°C is sufficiently well-tolerated, unlike at 29°C. To determine if the lethality due to haploinsufficiency was the consequence of an underlying deficit in DL-mediated patterning, we turned our attention to the cuticles of first instar larvae (Fig. 3c1). Cuticles with wild-type pattern were designated as Class 1, those with mild head defects as Class 2 and those with a severe phenotype, reminiscent of dorsalized, D3 embryos as Class 3. 50% of *dl^WT^/Df* embryos, in contrast to 87% of *dl^SCR^/Df* embryos appear as Class 1 (Fig. 3, c2–c3 and d), concurrent with the percentage of embryos that hatch. Forty-seven percentage of *dl^WT^/Df* embryos showed Class 2 phenotypes that were drastically reduced in *dl^SCR^/Df*, to 11%. A small fraction of embryos (∼3% of *dl^WT^/Df* and ∼2% of *dl^SCR^/Df*) showed a more severe Class 3 phenotype (Fig. 3, c2″*–*c3″). The rescue of embryonic lethality appeared to be a direct result of Class 2 embryos transitioning to normal, Class 1 embryos in the presence of the *dl^SCR^* allele. Our findings demonstrate that the *dl^SCR^* allele alleviates temperature-dependent haploinsufficiency, rescuing developmental patterning.

### DL^SCR^ supports the developmental program under haploinsufficient conditions

**Fig. 4.**
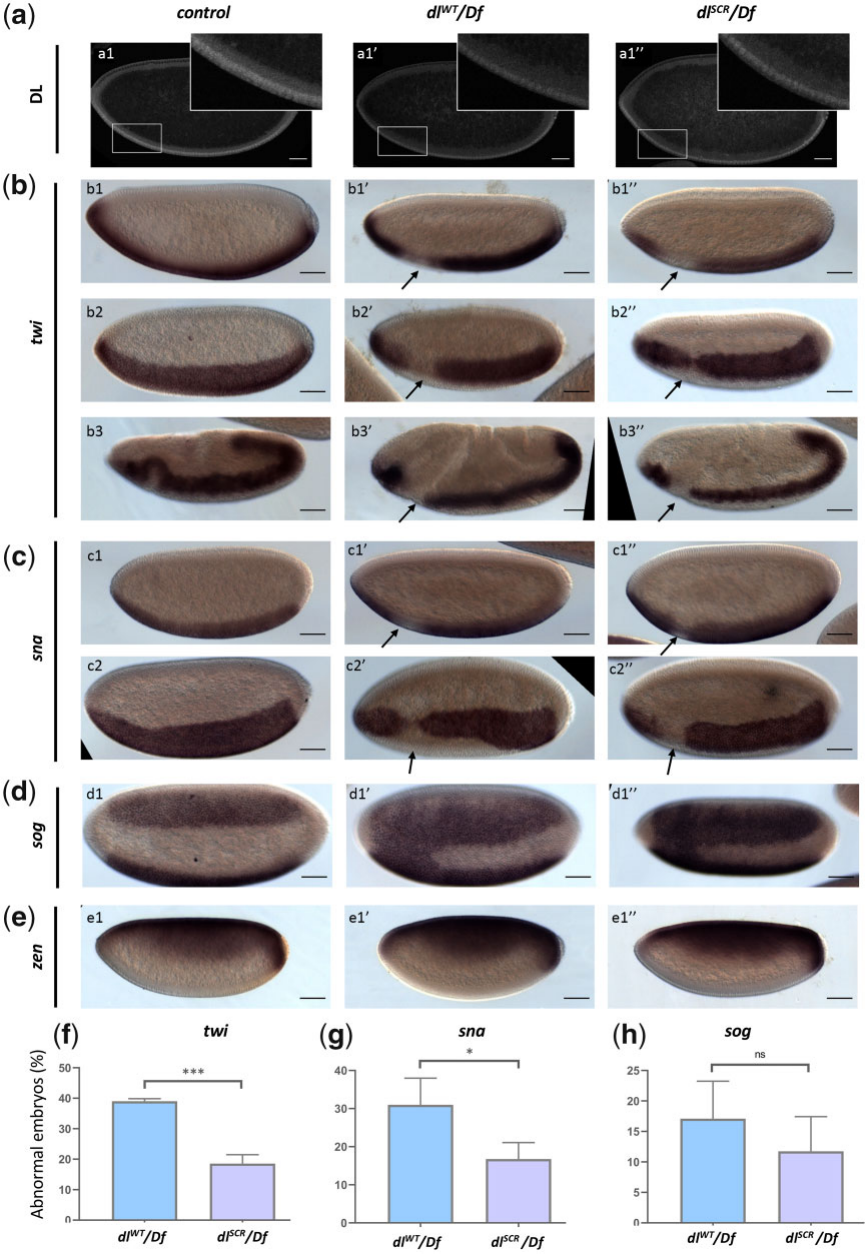
DL activity is altered in the SUMO-deficient mutant. The DL gradient was visualized with a DL antibody in *control*, *dl^WT^/Df* and *dl^SCR^/Df* embryos (a). Insets represent a zoomed-in view of the presumptive cephalic furrow in the ventral region. In situ hybridization images of stage 5 embryos probed with digoxigenin-AP-labeled antisense RNA probes against *twi* (b), *sna* (c), *sog* (d), and *zen* (e) are shown (b3–b3″ are stage 7 embryos, an exception). Embryos are oriented with the anterior side to the left and ventral side down (b1–b1″; b3–b3″; c1–c1″; e1–e1″), or tilted toward the reader (b2–b2″; c2–c2″; d1–d1″), for control (b1–e1), *dl^WT^/Df* (b1′–e1′), and *dl^SCR^/Df* (b1″–e1″). Arrows indicate a narrowing or an absence of the *twi* (a) and *sna* (b) pattern at the region of the presumptive cephalic furrow. d1′–d1″) A fusion of the *sog* gradient near the ventral cephalic region. Embryos showing a deviation from the normal pattern (narrowing/absence/fusion) for *twi*, *sna*, and *sog* were plotted as a percentage of total stained embryos, for the *control*, *dl^WT^/Df*, and and *dl^SCR^/Df* (f–h). Approximately 50 embryos were scored in each technical replicate, across 3 technical replicates. Data represented as mean ± SEM, unpaired *t*-test, (ns) *P > *0.05, (***) *P < *0.001, (*) *P < *0.05.

The nuclear DL gradient was similar between *dl^WT^/Df* and *dl^SCR^/Df* in both sagittal (Fig. 4a) and transverse cross sections (Supplementary Fig. 3a). As compared to WT controls, a single copy of the *dl* allele (*dl^WT^/Df* or *dl^SCR^/Df*) in mothers led to shallow and broad gradients (Supplementary Fig. 3b) in embryos, consistent with earlier studies (Liberman *et al.* 2009; Reeves *et al.* 2012; Ambrosi *et al.* 2014; Carrell *et al.* 2017; Al Asafen *et al.* 2020), though the differences are statistically insignificant. The *dl^WT^/Df* or *dl^SCR^/Df* gradients were also similar to each other (Supplementary Fig. 3, b–d). On the ventral and lateral sides of the embryo, nuclear DL activates 50–70 genes, e.g. *twi*, *sna*, *rhomboid (rho)*, *brinker (brk)*, and *short gastrulation (sog)*, while a smaller number, such as *decapentaplegic* (*dpp)* and *zen* are transcriptionally repressed. *twi* is one of the earliest targets of DL to be activated in the ventral region in the wild-type embryo (Fig. 4b1) (Jiang *et al.* 1991). In situ hybridization indicated that a large fraction (∼40%) of embryos laid by *dl^WT^/Df* mothers deviate from normal *twi* patterning (Fig. 4b2), and we observed a drastic reduction or complete disruption of *twi* expression in the regions that intersect with the presumptive cephalic furrow, in stage 5 embryos (Fig. 4, b1′–b2″), when compared to wild-type embryos (Fig. 4, b1–b2). We refer to this region (arrow), as the DL “weak activation region” or “WAR.” The lack of *twi* activation is not transient and persists even at later stages of germ band extension (Fig. 4, b3′ vs. b3). We observed similar defects in embryos derived from *dl^SCR^/Df* females (Fig. 4, b1″, b2″, and b3″), but their numbers were dramatically reduced in comparison to *dl^WT^/Df* (Fig. 4f). The DL gradient in *dl^WT^/Df* and *dl^SCR^/Df* embryos (Fig. 4a, insets) is uninterrupted in the WAR in all embryos, pointing to a local failure of DL mediated activation rather than reduced or lack of expression of DL, in the WAR region.

DL and Twi work synergistically to activate *sna* which is critical for mesoderm specification. Haploinsufficiency of *dl* also manifests as a severe loss or absence of *sna* at the WAR, closely mirroring the defects in *twi* expression in *dl^WT^/Df* and *dl^SCR^/Df* embryos (Fig. 4, c1–c2″). While ∼30% of *dl^WT^/Df* embryos appear defective in *sna* expression, only ∼15% of *dl^SCR^/Df* embryos display *sna* abnormalities (Fig. 4, c1–2, c1′–2′, c1″*–*2″, and g). In ∼10–15% of *dl^WT^/Df* and *dl^SCR^/Df* embryos (Fig. 4, d1′, d″, and g), the *sog* gradient is expanded in the WAR, allowing the 2 lateral *sog* stripes to fuse ventrally (Fig. 4, d1′ and d1″). The expansion of *sog* in the ventral domain is a direct consequence of the weaker expression of *sna/*Sna in the WAR. *zen*, repressed in the ventral and lateral regions by DL and expressed only at the dorsal side of the embryo remained unperturbed in the haploinsufficient embryos of both *dl^WT^* and *dl^SCR^* (Fig. 4, e1–e1″). This was in stark contrast to the failure of DL-mediated activation, suggesting that DL-mediated repression was not influenced by the SUMOylation status of DL, even under haploinsufficient conditions.

Thus, the *dl^SCR^* allele rescues the failure of activation in the WAR for a large fraction of haploinsufficient embryos. The data described in this section argue for a role for SUMO conjugation of DL in regulating activation of DL target genes, especially *twi* and *sna*, in the WAR. SUMO conjugation-competent embryos (wild type) have a high failure rate for activation of *twi* and *sna* in the WAR under haploinsufficient conditions, when compared to DL^SCR^. The in situ data presented in this section are excellent for discerning spatio-temporal changes in the expression of DL target genes. What is yet unanswered is the effect of DL^SCR^ on the levels of transcripts of DL targets, especially in conditions of haploinsufficiency. For this, we turned to quantitative mRNA measurements using RNA sequencing, described in the next section.

### DL^SCR^ is a stronger transcriptional activator than DL^WT^ under conditions of haploinsufficiency

**Fig. 5.**
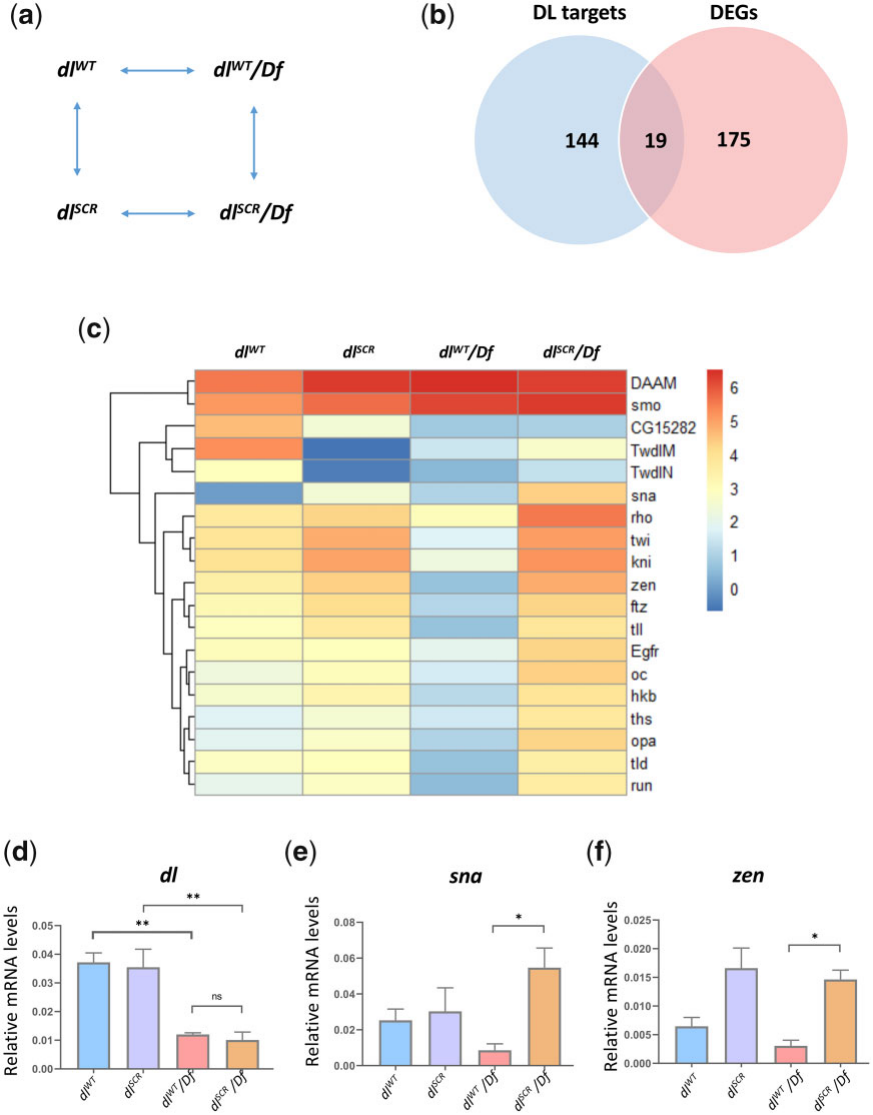
DL^SCR^ displays higher transcriptional activity. Maternal genotypes of the embryos used for the 3′ RNA-seq analysis and their pairwise comparison to obtain DEGs is presented in (a). Genes that were identified as direct targets of DL from published literature and DEGs across all the conditions are represented as a Venn diagram in (b). The subset of DEGs with known binding sites for DL is represented as a heatmap, for *dl^WT^*, *dl^SCR^*, *dl^WT^/Df* and *dl^SCR^/Df* embryos at 29°C in (c). LogCPM values are plotted. (d–f) Relative mRNA expression levels of *dl*, *sna* and *zen* transcripts, respectively, measured by qRT-PCR analysis, for 0–2 hr embryos laid by mothers of the indicated genotypes at 29°C. *N* = 3, mean ± SEM, ordinary 1-way ANOVA, (ns) *P > *0.05, (**) *P < *0.01, (*) *P < *0.05

To obtain a global picture of the transcriptional activity of DL^SCR^, we conducted a quantitative 3′ RNA sequencing experiment on embryos laid by *dl^WT^* and *dl^SCR^* mothers, as well as embryos derived from *dl^WT^/Df* and *dl^SCR^/Df* mothers. The experiment was conducted at 29°C for embryos aged 0–2 hr after egg lay, to capture possible quantitative differences in activation of DL target genes on account of maternal DL. Details of the methodology can be found in Materials and Methods. Overall, across the 4 genotypes (*dl^WT^*, *dl^SCR^*, *dl^WT^/Df*, and *dl^SCR^/Df*; Fig. 5a) studied, 194 genes are differentially expressed (−0.58 >log_2_ fold change > 0.58, at FDR < 0.1; ST-1), visualized as a heat map (Supplementary Fig. 4a; ST-1). A gene ontology analysis confirmed significant enrichment of genes encoding proteins involved in DNA-binding and embryonic developmental processes (Supplementary Fig. 4b). Of these, we focused on genes that are bona-fide DL targets (*n* = 163, Fig. 5b), collated from studies on DV mutants using microarray chips, ChIP-chip analysis and bioinformatics studies (Markstein *et al.* 2002; Stathopoulos *et al.* 2002; Zeitlinger *et al.* 2007). Of the 194 DEGs in our 3′ RNA sequencing experiment (ST-1E), 19 are known DL targets (Fig. 5b), and we generated a heat map to visualize the differences in gene expression across the 4 genotypes (Fig. 5c).

As expected, analysis of the transcript levels (represented as log_CPM_ values) clearly indicates that genes like *twi*, *knirps (kni)*, *zen*, *fushi tarazu (ftz)*, *tailless (tll)*, and *tolloid (tld)* show lowered transcripts in *dl^WT^/Df* compared to *dl^WT^*. A reduction in the dose of *dl* in haploinsufficient embryos leads to reduced transcriptional activation of DL target genes (Fig. 5c). Exceptions include *Disheveled Associated Activator of Morphogenesis (DAAM)* and *smoothened (smo)*, whose transcript levels go up significantly, with decreased *dl* levels. When compared to *dl^WT^*, *dl^SCR^* did not display statistically significant differences for *twi*, *kni*, *zen*, *ftz*, and *tll*, nor did we observe significant differences between *dl^SCR^/Df* and *dl^SCR^* (Fig. 5c). Intriguingly, 14 DL target genes are significantly upregulated in *dl^SCR^/Df* compared to *dl^WT^/Df* (Fig. 5c andSupplementary Fig. 4c) suggesting that for DL^SCR^, the lowered dose of DL in the haploinsufficient embryos is compensated for by higher transcriptional activation of critical DL target genes. These genes include DV patterning targets such as *twi, sna, zen* and *rho*, and anterio-posterior (AP) target genes such as *kni* and *huckebein (hkb)* (Supplementary Fig. 4c). The *dl*, *sna* and *zen* transcript levels were further independently assessed in a qRT-PCR experiment (Fig. 5, d–f). *dl* transcripts themselves are comparable across *dl^WT^/Df* and *dl^SCR^/Df*, downregulated by ∼50% compared to the control (Fig. 5d), indicating that *dl* mRNA levels are not affected in *dl^SCR^*. *Sna* and *zen*, however, show 6–8-fold higher transcript levels (Fig. 5, e and f) in *dl^SCR^/Df*, in agreement with our 3′ mRNA sequencing data (Fig. 5c). *zen* is a target for DL-mediated repression, but in the absence of expansion of the *zen* expression domain, the transcript data suggests that a transcriptional activator responsible for switching on *zen* may be indirectly influenced by DL.

The greater rate of hatching and survival of *dl^SCR^/Df* compared to *dl^WT^/Df* is possibly a function of increased, compensatory transcription of DL targets and the previously described rescue of activation in the WAR. SUMOylation of DL thus plays a global role in decreasing transcription of DL targets in general and in addition, has an important role in the WAR for activation of *twi/sna*. The higher activation of DL target genes in *dl^SCR^/Df* leads us to hypothesize (see *Discussion*) that SUMO conjugation of DL may be part of a negative feedback loop to curtail transcription of DL targets.

### SUMO restrains DL activity in the larval immune response

**Fig. 6.**
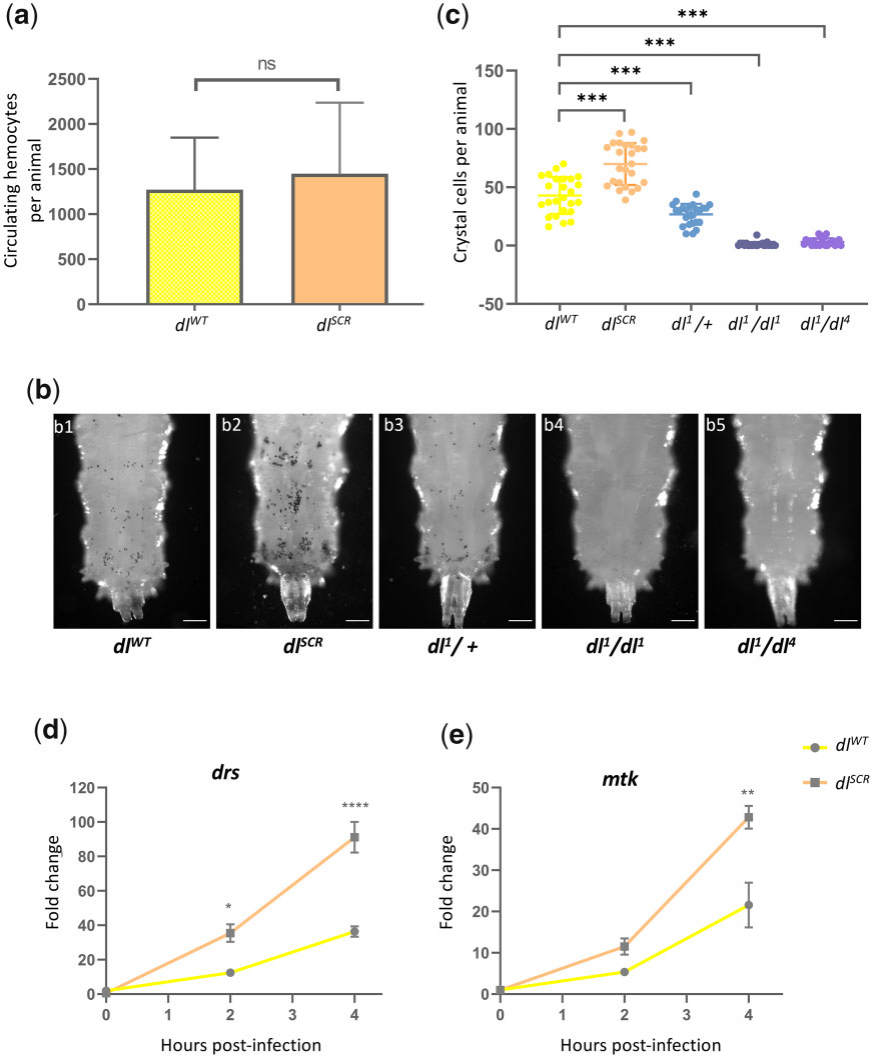
DL^SCR^ is a robust immune effector in the larva. Total circulating hemocytes for *dl^WT^* and *dl^SCR^* are plotted as a bar graph in (a). *N* = 3, Mean ± SEM, unpaired *t*-test, (ns) *P > *0.05. Crystal cells in the third-instar larva were observed under a bright-field microscope, for the genotypes indicated in (b). The last 3 posterior segments were imaged with the dorsal side facing the viewer. The number of crystal cells in the posterior segments was counted per animal for each genotype and is represented in (c). *N* = 3, mean ± SEM, ordinary 1-way ANOVA, (***) *P < *0.0001. Transcript levels of Toll-responsive AMPs—*drs* and *mtk* (d) analyzed by qRT-PCR are plotted for the control and *dl^SCR^*. Data were collected at 0, 2, and 4 hr after septic injury with the Gram-positive pathogen *S. saprophyticus*, in the third instar larvae*. N* = 3, mean ± SEM, 2-way ANOVA, (*) *P < *0.05, (**) *P < *0.01, (****) *P < *0.0001. Data are representative of at least 8 larvae per replicate, across 3 independent biological replicates.

Earlier, Bhaskar *et al.* (2002) found that DL^SCR^ is a better transcriptional activator, assessed by luciferase reporter activity on artificial promoter clusters in S2 cells. Since S2 cells are hematopoietic in origin, we reasoned that SUMO conjugation of DL may also influence the immune response in animals. The *dl^SCR^* line allows us to conduct similar experiments in the larvae, with twin advantages over S2 cells of working in the animal and the absence of confounding wild-type DL in the background. Septic injury by Gram-positive bacteria (or fungal infections) leads to the upregulation of *dl* transcripts and translocation of DL to the nucleus in the larval fat body, a major effector site of the humoral response (Reichhart *et al.* 1993; Lemaitre *et al.* 1995; Manfruelli *et al.* 1999). Similar behavior was seen for Dif (Ip *et al.* 1993; Meng *et al.* 1999; Govind 1999; Hoffmann 2003). Gain-of-function mutants in Toll/NF-κB signaling display an over-proliferation of hemocytes (Qiu *et al.* 1998; Matova and Anderson 2006). Studies have also implicated Dif and DL as effectors causing melanotic tumors when constitutively nuclear (Huang *et al.* 2005; Chiu *et al.* 2005). We reasoned that if DL activity was indeed affected in *dl^SCR^*, it might influence blood cell numbers, but the number of circulating plasmatocytes remained unchanged in *dl^SCR^* mutants (Fig. 6a), in uninfected conditions. We also looked at crystal cells (Fig. 6b1), a platelet-like population of cells important for melanization and wound healing (Vlisidou and Wood 2015), visualizing the activation of the melanization cascade in response to heating/boiling of larvae (Rizki and Rizki 1959; Lanot *et al.* 2001). To our surprise, *dl^SCR^* larvae showed a marked increase in crystal cell numbers (Fig. 6, b2 and c) in comparison to the wild type (Fig. 6, b1 and c). Also striking was the near absence of crystal cells in *dl^1^/dl^1^* animals (Fig. 6, b4 and c), defined in literature as a null allele (Isoda *et al.* 1992), which we find is *dl^S317N^* (Supplementary Fig. 5). A severe reduction in crystal cell number is also evident when another null allele of *dl*, *dl^4^* is used in a trans-allelic combination with *dl^1^* (Fig. 6, b5 and c). Crystal cell numbers were intermediate when only 1 functional allele of *dl* was present (Fig. 6, b3 and c). A similar dose-dependent response of crystal cell number is observed when *dl^SCR^* is compared with *dl^1^/dl^SCR^* or *dl^4^/dl^SCR^* larvae (Supplementary Fig. 6). DL may regulate phenoloxidase activity (Bettencourt *et al.* 2004) or determine crystal cell fate, which is known to be specified by interactions between Serpent, Lozenge, and U-shaped (Banerjee *et al.* 2019). Though evidence for DL/Srp co-operativity in determining hematopoietic cell-fate is lacking, we do see a direct correlation between melanized cells and levels of DL activity, with *dl^null^* animals lacking melanization and *dl^SCR^*, our presumptive transcriptionally active allele (*dl^SCR^*) showing the highest levels.

We also monitored the temporal expression of AMPs upon septic injury in *dl^WT^* and *dl^SCR^*, to gauge the humoral response. Third instar larvae were infected with the Gram-positive bacteria *S.* *saprophyticus* and the induction of Toll-specific AMPs *drosomycin* (*drs*) and *metchnikowin* (*mtk*) was analyzed using qRT-PCR at 2 and 4 hr postinfection. DL^SCR^-induced AMPs to a 2-fold higher level, in comparison to DL^WT^ (Fig. 6, d and e). The effect was more prominent at 4 hr postinfection, with both *drs* and *mtk* showing significantly higher expression (Fig. 6, d and e). These above results agree with our thesis that DL^SCR^ is a stronger transcriptional activator and are also in agreement with S2 cell data published previously (Bhaskar *et al.* 2002).

### Dynamics of nuclear import of DL^SCR^ in the larval fat body

**Fig. 7.**
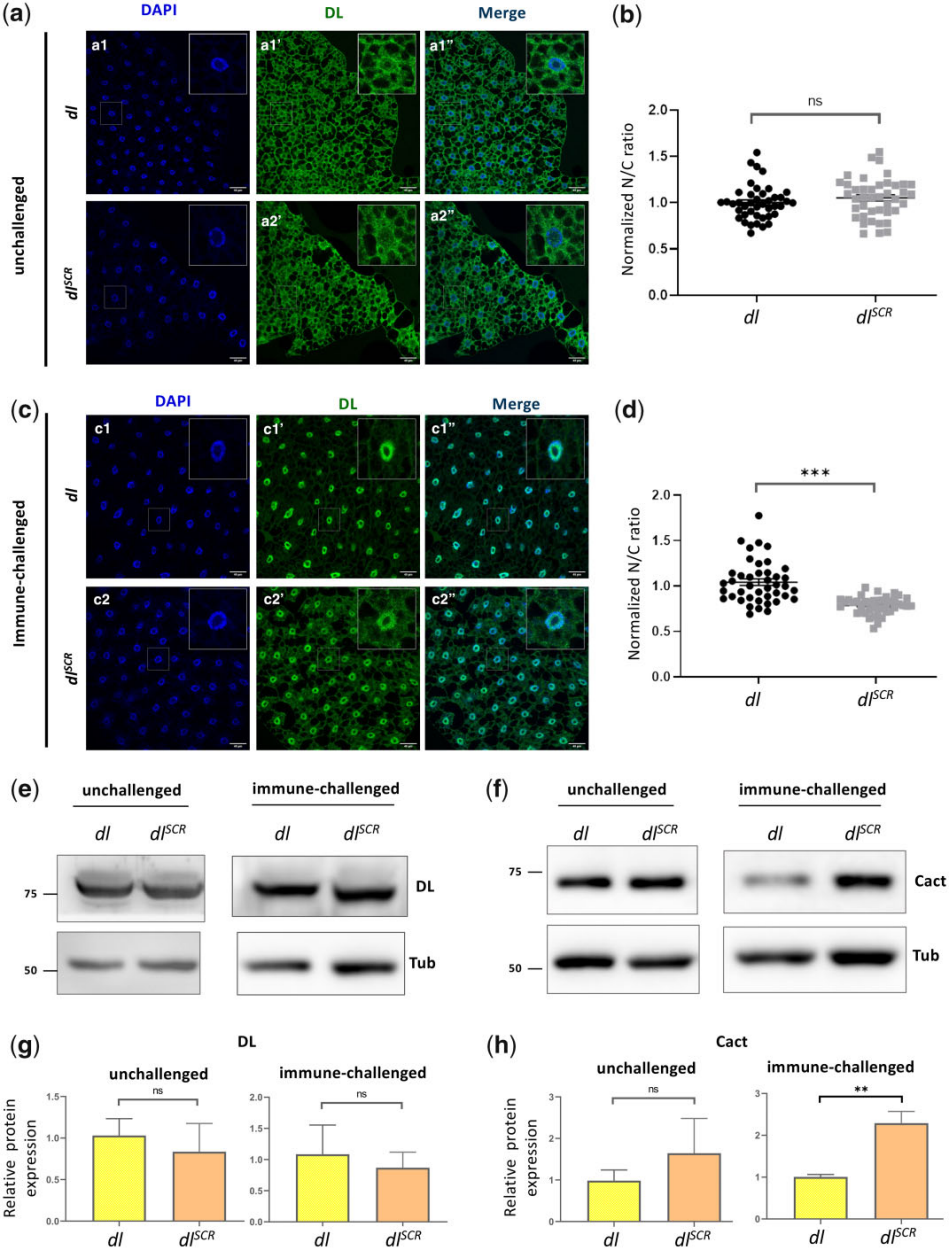
DL^SCR^ is responsive to Toll signaling in the larval fat body. DL is visualized via antibody staining (green), in the uninfected state (a) and infected state (c). Nuclei are labeled with DAPI (blue). Merged images (a1″) and (a2″) indicate uniform distribution in fat body cells. DL levels in the cytoplasm and nucleus were quantified and plotted as a nuclear/cytoplasmic (N/C) ratio for control and mutant (b). The N/C ratio was calculated for >40 cells in at least 5 fat bodies, across 3 independent replicates. Individual values are represented on a scatter plot, bar denotes mean ± SEM, statistical significance inferred by unpaired *t*-test, (ns) *P > *0.05, (***) *P < *0.001. DL predominantly partitions to the nucleus 60 min after infection with *S. saprophyticus*, evident in merged images (c1″) and (c2″). N/C ratio was quantified and plotted for *dl^SCR^* and *dl^WT^* (d), as in (b). Protein levels of DL and Cact in the unchallenged and immune-challenged fat body were determined by a western blot, shown in (e) and (f), respectively. Protein levels were normalized to the loading control (tubulin) and quantified, represented as relative expression levels below the respective blots for *dl^WT^* (yellow bar) and *dl^SCR^* (orange bar) (g and h). *N* = 3, bar chart represents mean ± SEM, statistical significance calculated by unpaired *t*-test, (ns) *P > *0.05, (***) *P < *0.001.

In the context of the embryo, DL import appeared to be normal in the SCR allele, with no evidence that supported a change in the DL DV gradient. The primary difference between the DL^WT^ and DL^SCR^ was seen in haploinsufficient conditions, where the overall DL-mediated activation was weaker (Fig. 5d) due to low concentrations of DL in the nucleus. In addition to global lowering of transcripts of DL target genes, a complete loss of activation of *twi* was seen in a specific spatiotemporal region, the WAR. In *dl^SCR^* embryos, the absence of DL SUMOylation appeared to suppress the weakened transcriptional activation. This is in line with the higher transcriptional activation seen in S2 cells and also in the larval fat body for DL^SCR^. Furthermore, we measured the extent of nuclear import in the fat body of larvae. DL^SCR^ is retained in the cytoplasm in un-infected larvae, similar to the wild type (Fig. 7, a1″*–*a2″). Intensity-based quantitation of the nuclear to cytoplasmic ratio (N/C) is also comparable (Fig. 7b), indicating that DL’s SUMOylation is dispensable in retention of the DL/Cact complex in the cytoplasm, in the absence of active Toll signaling.

We next monitored the status of DL in the fat body, 60 min after an immune challenge with the Gram-positive pathogen *S.* *saprophyticus*. DL^SCR^ is competent in its ability to enter the nucleus (Fig. 7c2″), at par with the wild type. Paradoxically, the normalized N/C ratio for DL appears to be lower than wild-type, in the *dl^SCR^* mutants (Fig. 7d), indicating that relative to wild type, more DL is retained in the cytoplasm or that less DL is imported to the nucleus. The images (Fig. 7c) suggest that the former is true, with a larger fraction of DL^SCR^ apparently retained in the cytoplasm compared to DL animals. The retention of DL^SCR^ in the cytoplasm can be due to many reasons. There could be (1) an increased affinity of DL^SCR^ for Cact (2) a decreased rate of nuclear import or an increased rate of nuclear export, or (3) an increase in Cact concentration in the cytoplasm, which would, in turn, stabilize and retain DL.

Western blots suggest that both DL^WT^ and DL^SCR^ are expressed at similar levels in the fat body, both in unchallenged and *S. saprophyticus*-challenged larvae (Fig. 7e), potentially ruling out the K382 residue as a site for ubiquitination that specifically affects DL stability. Intriguingly, Cact levels were found to be higher in infected conditions in *dl^SCR^* animals (Fig. 7f). The *cact* promoter/enhancer region has binding sites for DL, allowing DL to positively regulate Cact levels (Nicolas *et al.* 1998; Paddibhatla *et al.* 2010). Though there are multiple possibilities, we hypothesize that the DL^SCR^ allele, being more active, leads to increased transcription of *cact*. Excess Cact in the cytoplasm, in turn, leads to efficient retention of DL.

### A mathematical model to investigate the activity of SUMOylated DL

Experimental data from S2 cells, the embryo and larvae, all suggest that UnSUMOylated DL (DL^U^) is a stronger transcriptional activator with SUMOylation of DL being a mechanism to attenuate DL mediated activation. Since SUMOylated DL (DL^S^) levels are in the range 1–5% of total DL (Bhaskar *et al.* 2000; Smith *et al.* 2004), as estimated by the ratio of SUMOylated/unSUMOylated species on western blots, it is difficult to examine the activity of DL^S^ experimentally. We have attempted to gain additional insight into roles for the DL^S^ species by generating a mathematical model and numerically evaluating the effect of DL SUMOylation.

**Fig. 8.**
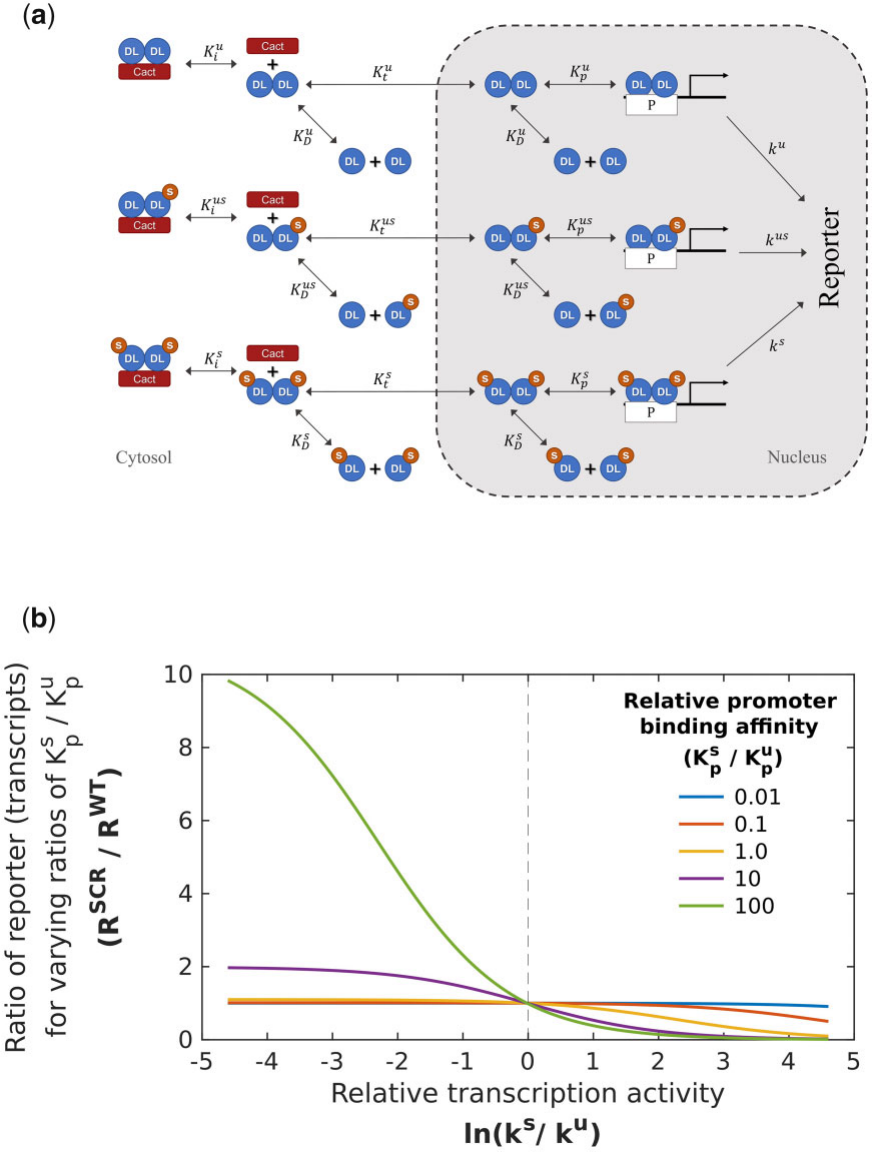
Mathematical model to understand roles for SUMOylated DL. Processes included in the mathematical model (a) UnSUMOylated (DL^U^) and SUMOylated DL (DL^S^) reversibly dimerize to form homo- and heterodimers (equilibrium constant $K_{D}$) which are inhibited by Cact in the cytoplasm through reversible binding (equilibrium constant $K_{i}$). Dimers partition into nucleus ($K_{t}$) where they bind to the promoter P (binding constant $K_{p}$). Bound promoter catalyzes first-order reporter expression with rate constants denoted by k. Parameters for reactions involving the 2 homodimers and the heterodimer are represented by superscripts u, s, and us, respectively. b) Simulating the effect of SUMOylation of DL on transcription of DL target genes. Ratio of reporter expression levels (R^SCR^/R^WT^) is obtained by solving Supplementary Equations (23)–(28) with parameters listed in Supplementary SI-2. The rate of transcriptional activation by the DL^U^ homodimer ($k^{u}$) bound to the promoter is kept constant, and the ratio of reporter expression is calculated when the rate ($k^{s}$) for promoter-bound DL^S^ dimers is varied 2 orders of magnitude from this level. The process is repeated for different values of relative promoter binding affinity ($K_{p}^{s}/K_{p}^{u}=\;0.01,\;0.1,\;1,\;10$, and 100). The reporter expression levels for *dl^SCR^* are greater than the corresponding WT levels when the relative transcription activity is lower ($k^{s}/k^{u}<\;1$) for DL^S^ and there is tighter binding of SUMOylated dimers to the promoter ($K_{p}^{s}/K_{p}^{u}>1$). Other parameter ratios ($K_{D}^{s}/K_{D}^{u}$, $K_{t}^{s}/K_{t}^{u}$, and $K_{i}^{s}/K_{i}^{u}$) are kept at 1.

To computationally explore potential causes for the observed increase in reporter expression in *dl^SCR^* compared to *dl^WT^*, we have incorporated processes involved in DL signaling, such as reversible dimerization, Cact-binding, nuclear partitioning, and binding/activity at promoter sites, in our mathematical model (Fig. 8a and Supplementary SI-1). The rates and therefore the equilibrium constants of the reactions defined in our model may be different for DL^S^ and DL^U^. The rate of reporter expression (Fig. 8a), which is equivalent to measuring DL-mediated transcription, is assumed to depend on the fraction of dimers bound to the promoter site, with a specific rate depending on whether the dimers exist as DL^S^ or DL^U^ homodimers. In our model, we assume that the reactions are in a (pseudo) steady state, i.e. we assume that there is no change in total DL and total Cact levels due to expression or degradation.

With a steady-state assumption, information on equilibrium constants, and not the individual reaction rate constants is required for every reversible process. These parameters were approximated from reported values for the same or similar proteins (Supplementary SI-2) from mammalian (Tay *et al.* 2010) and insect (Kanodia *et al.* 2009; Carrell *et al.* 2017; Ramsey *et al.* 2019; Al Asafen *et al.* 2020) systems. These parameters were used for reactions involving DL^U^, and values for the SUMOylated DL reactions were varied a hundred-fold relative to the starting value. A mass balance on each component resulted in 18 algebraic equations and 4 conservation equations [Supplementary Equations (1)–(22)] that were simplified and numerically solved (*Materials and* *Methods* and Supplementary SI-1). For a given set of parameters, the steady state reporter expression is calculated for WT and SCR conditions (i.e. 5% SUMOylated and 0% SUMOylated DL respectively).

The ratio **(**$R^{\mathrm{SCR}}/R^{WT}$**)** of the steady state reporter expression for *dl^SCR^* and *dl^WT^* thus calculated is represented on the *y*-axis of Fig. 8b. A value of 1 indicates that the reporter expression is unchanged in the *dl^SCR^* and *dl^WT^*, and values greater than 1 indicate that the reporter expression is greater in the SCR mutant, as is observed experimentally. Hence, we seek to computationally identify conditions that lead to a value greater than 1 for the relative reporter expression. To this end, we repeat the calculations of relative expression ratio for multiple values of the equilibrium constant corresponding to the SUMOylated species for 1 process **(**such as specific transcriptional activity $k^{s}$), while keeping the equilibrium values constant for the corresponding process with DL^U^**(**such as $k^{u}$**)**. This is represented in the *x*-axis of Fig. 8b. Here, the specific activity ($k^{s}$) corresponding to bound DL^S^ is varied over 2 orders of magnitude relative to $k^{u}$. For each value of $k^{s}$/$k^{u}$ from 0.01 to 100 *(x*-axis value from −4.6 to 4.6 corresponding to ln(0.01) and ln(100), respectively), the reporter expression ratio is calculated and plotted on the *y*-axis. This calculation of relative reporter expression as a function of specific activity is repeated at 5 different promoter binding affinity values ($K_{p}^{s}$) for dimers containing DL^S^**(**colored lines plotted in Fig. 8b corresponding to values of $K_{p}^{s}/K_{p}^{u}$ from 0.01 to 100**)**. The parameters for the other processes involving DL^S^ are assumed to be the same as the parameters for the corresponding processes with DL^U^. We observe that when SUMOylation does not affect the specific activity **(**x=0 on the graph in Fig. 8b, i.e. $k^{u}=k^{s},\;\ln\left(k^{s}/k^{u}\right)=0$**)**, the relative reporter expression ($R^{\mathrm{SCR}}/R^{WT}$) remains almost constant at 1, irrespective of the change in binding affinity by multiple orders of magnitude. Only when $k^{s}<k^{u}$, i.e. $\ln\left(k^{s}/k^{u}\right)<0$, does the relative reporter expression become greater than 1. Note that this increase is seen only when there is a substantial enhancement in the promoter-binding ability of dimers containing DL^S^**(**i.e. $K_{p}^{s}/K_{p}^{u}\geq 10$). Further calculations show that while decrease in transcriptional activity of promoter-bound dimers containing DL^S^ compared to the activity of bound DL^U^ homodimers ($k^{s}<k^{u}$) is necessary, another factor that enhances the fraction of bound SUMOylated DL dimers, such as greater binding ability (Fig. 8b) or greater extent of partitioning to the nucleus (Supplementary Fig. 7, middle row, right panel) or lesser sequestration by Cact (Supplementary Fig. 7, bottom row, right panel), is required for a substantial increase in the reporter expression for SCR mutants. Simulating combinations of changes due to SUMOylation in other processes, keeping the transcriptional/reporter activity of bound DL^S^ and DL^U^ unchanged **(**i.e. $k^{u}$=$k^{s}$), does not change the relative expression ratio substantially (Supplementary Fig. 7, left-side, all panels). These results indicate that increased expression in *dl^SCR^* mutants is likely to be associated with ($k^{s}<k^{u}$), or a reduced activity of bound DL^S^. This may also explain the ability of DL^SCR^ in the embryo to rescue the effect of *dl* haploinsufficiency. Lower total DL due to the loss of an allele may lead to lower activation, which increases to near-WT levels when SUMOylation is abrogated.

## Discussion

Comprehensive proteomic studies across species have led to the identification of SUMOylated proteins (Wykoff and O'Shea 2005; Handu *et al.* 2015; Hendriks and Vertegaal 2016; Pirone *et al.* 2017; Hendriks *et al.* 2018). One class of proteins studied in detail is TFs (Verger *et al.* 2003), with ∼50% being SUMOylated in humans (Hendriks *et al.* 2017). Upon SUMOylation, changes in the transcriptional output of TFs can be brought about by alterations in DNA-binding, eviction from the chromatin, or a re-shaping of their protein-interaction landscape (Ouyang and Gill 2009; Raman *et al.* 2013; Wotton *et al.* 2017; Rosonina *et al.* 2017; Rosonina 2019). Amongst the well-studied TFs is the NF-κB family (Kracklauer and Schmidt 2003; Mabb and Miyamoto 2007). SUMOylation of NF-κB was first demonstrated in *Drosophila*, for DL (Bhaskar *et al.* 2000). In mammals, RelA undergoes SUMOylation after TNFα stimulation, aided by the E3 ligase PIAS3. The authors also observe, interestingly, that only the DNA-bound form of RelA is SUMO-modified (Liu *et al.* 2012) and acts as a repressor. RelB, another NF-κB family TF, is also negatively regulated by SUMOylation, though its DNA binding remains unchanged (Leidner *et al.* 2014).

**Fig. 9.**
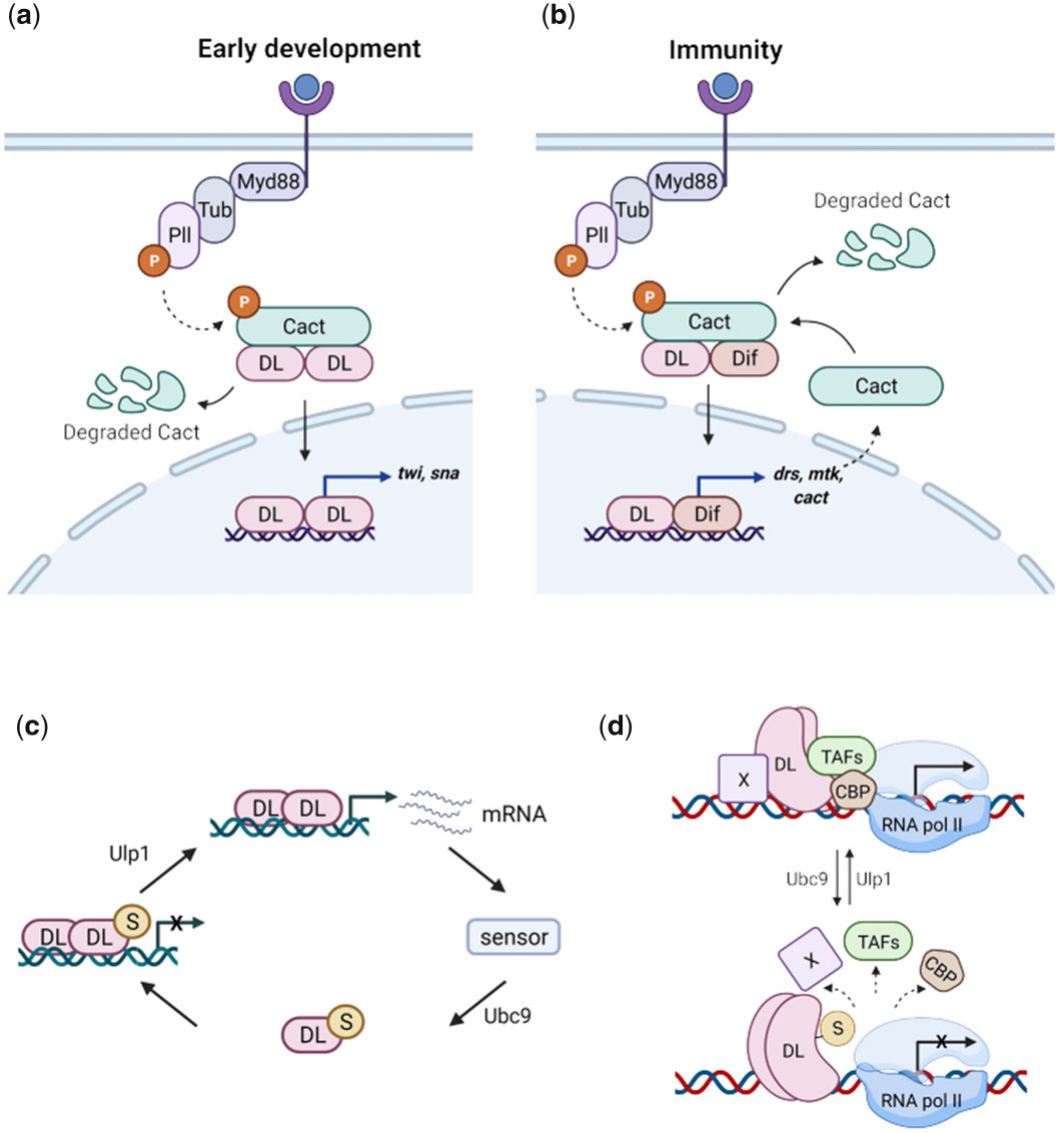
DL SUMOylation attenuates Toll signaling, acting via a feedback circuit. We suggest the following model for dampening of the Toll signal upon DL SUMOylation. When Toll signaling is initiated, DL migrates to the nucleus, and activates target genes, in both the developmental and immune contexts (a and b, respectively). UnSUMOylated DL activates transcription of DL-target genes. Once optimum levels of target transcripts are reached, or under conditions of stress, SUMOylation of DL is triggered, through as yet unknown mechanisms, curtailing excessive transcription (c). Transcriptional activity of DL may be regulated via conserved interactions with CBP and/or TAFs in association with a protein “X,” most likely GATA-factor Srp in immunity or bHLH proteins like daughterless/achaete-scute, in early development (d). SUMOylation of DL may perturb these interactions, attenuating transcription.

In *Drosophila*, the Toll/NF-κB cascade has been best studied in 2 diverse contexts, in DV patterning (Fig. 9a) and in the immune response (Fig. 9b). In early development, where perturbations to the DL gradient could derail DV patterning, we do not see any effect of lack of SUMOylation on DL activity. Increase in the transcriptional activity of DL^SCR^ in the embryo becomes apparent only in conditions of haploinsufficiency (*dl^SCR^/Df*), where DL target genes are, surprisingly, activated at wild-type levels. This enhanced transcriptional activation with reduced DL dosage leads us to suggest that SUMO conjugation may be linked to a negative-feedback loop (Fig. 9c), where transcription of DL-target genes is sensed by a hypothetical sensor, that triggers SUMOylation of DL by Ubc9. In our model, DL^S^, when bound to the promoter, would block activation by DL^U^ and attenuate DL signaling. The sensor would ideally sense transcripts of DL target genes, as shown for miRNAs (Li *et al.* 2021). Under conditions of haploinsufficency (*dl^WT^/Df*) at 29°C, DL^U^ activates target genes, with DL^S^ dampening the response, leading to lowered levels of transcripts. In the case of *dl^SCR^/Df*, the circuit (Fig. 9c) is broken, with Ubc9 unable to SUMOylate DL^SCR^. Here, DL-mediated activation cannot be dampened, leading to higher levels of target transcripts.

An interesting feature of haploinsufficiency in the embryo is the WAR. This phenomenon goes beyond a generic lowering of *twi* activation and is possibly related to the modulation of a physical interactor of DL (Supplementary Fig. 8). Candidates include Daughterless, Achaete-scute complex, Nejire/CREB-binding protein and TBP-associated factors. Embryos laid by *nej^3^/+*; *dl^1^/+* mothers showed weaker *twi* expression and a lack of expression in the WAR (Akimaru *et al.* 1997). Similar effects were seen in eggs laid by *dl^1^/+*; *TAF_II_110/+* or *dl^1^/+*; *TAF_II_60/+* mothers (Zhou *et al.* 1998) as also *dl^8^/+*; *da^11B31^/+* mothers (Gonz**á**lez-Crespo and Levine 1993). We hypothesize that DL forms complexes with these proteins to activate *twi* and this complex formation is critical in the WAR (Fig. 9d). DL^U^ is efficient at interacting with one (or all) of these proteins whereas DL^S^ de-stabilizes the activation complex (Fig. 9d). Hence, in the *dl^SCR^* animal, the absence of DL^S^ leads to a robust activation of *twi* in the WAR, suppressing the lethality under conditions of haploinsufficiency.

Does our data suggest any role for DL in DV patterning under ambient conditions with normal DL concentrations? Since the developing embryo would face temperature fluctuations, DL^S^ could fine tune transcription rates and influence the robustness of the DL activity gradient. SUMO conjugation of DL would thus be a mechanism for developmental canalization, as hypothesized by Waddington (1959). High transcriptional activation by DL^U^, which could disturb DV patterning, would be dampened by DL^S^, allowing the embryo to maintain graded DL activity and complete the DV program successfully. In poikilotherms such as *Drosophila*, SUMO conjugation of DL would be a useful mechanism to buffer transcriptional activity against environmental perturbations and stochastic fluctuations, late in the cascade, specifically at the level of transcriptional activation.

In response to infection (Fig. 9b), *dl^SCR^* larvae exhibit an increase in the transcription of Toll-specific AMPs and show a higher number of crystal cells. *dl^SCR^* animals show 2–4-fold higher transcripts of *drs* and *mtk*, under infective conditions and a 2-fold increase in crystal cells, in the absence of infection. Again, as suggested earlier (Fig. 9c), SUMOylation of DL may be a mechanism to attenuate DL-mediated activation. Here, the GATA-family TF Serpent (Srp) may be an essential player. DL, Dif, and Relish are known to synergize with Srp (Petersen *et al.* 1999; Senger *et al.* 2004) in the larvae, and SUMOylation of DL may weaken or break these interactions. Srp and the RUNX-factor Lozenge (Lz) are critical for specifying crystal cell fate in embryonic and larval stages (Fossett *et al.* 2003). Our observation that *dl^null^* animals have few or no melanized crystal cells while *dl^SCR^* larvae have increased crystal cells may point to a hitherto unknown function of DL carried out in assistance with Srp/Lz. Further, in the larval fat body, nuclear partitioning of DL is affected in *dl^SCR^* animals in response to septic injury. Cact is more stable in the cytoplasm of *dl^SCR^* animals, and this would lead to retention of DL. Nevertheless, transcript levels of DL target genes are higher, leading us to hypothesize that *dl^SCR^* animals have higher *Cact* levels, and higher Cact levels could explain the enhanced retention of DL in the cytoplasm. However, the possibility of increased binding affinity of DL^U^ for Cact cannot be ruled out.

Our work further highlights the intricate fine-tuning that regulates signaling cascades. Toll signaling is modulated at multiple levels (Anderson 2000). Extracellular feedback exerted by serine hydrolase cascades serves as an initial checkpoint for receptor activation. Cact and WntD (Ganguly *et al.* 2005; Gordon *et al.* 2005) act as intracellular, cytoplasmic gatekeepers of DL activation. An additional phosphorylation step is necessary for the nuclear import of DL (Drier *et al.* 1999). Once in the nucleus, DL can interact with partner activators and co-repressors to calibrate the transcriptional output. Our data suggests an additional layer of control, within the nucleus, with SUMO conjugation as a means of keeping DL in check, downstream of its nuclear import. The SUMO conjugation machinery resides in the nucleus and this is the most probable site for SUMO-conjugation/deconjugation of DL. The SUMO conjugase Ubc9 is a physical interactor of DL (Supplementary Fig. 8 and Fig. 9c) and presumed to be placed proximal to the site of transcription (Bhaskar *et al.* 2000). The SUMO deconjugase Ulp1 may also be similarly localized (Anjum *et al.* 2013).

Interestingly, a previous study indicated that SUMO-conjugated DL showed an increased activation of target genes compared to wild-type DL, though considerably lower than DL^K382R^ (Bhaskar *et al.* 2002). The authors suggest that the presence of a synergy consensus (SC) motif at K382 recognized by a putative SC factor (SCF) that is recruited to DL^WT^ attenuates transcription. Both SUMOylation and the K382R mutation in DL are thought to abolish the interaction with the SCF, leading to higher transcriptional activation. In contrast, our data suggests that the fraction of DL^S^ in the WT animal acts as an impediment to transcription, while DL^SCR^ or DL^U^ are better transcriptional activators. A caveat of the previous study is the overexpression of Ubc9 being used as a proxy for increased DL SUMOylation. The overexpression of Ubc9 could influence the SUMOylation status of various other proteins, including DL interactors, indirectly affecting the transcriptional output of DL. The CRISPR-edited DL^K382R^ in our study allows us to unequivocally assign the phenotypic effects observed to a loss of Dorsal SUMOylation.

Since a very small proportion of total DL is SUMO conjugated, SUMOylation/deSUMOylation may be a dynamic process that defines the occupancy of “active” DL for transcription. Though we have assumed a value of 5% for the calculation in Fig. 8b, these results are qualitatively unchanged if lower (1%) or higher (10%) SUMOylated DL is assumed (Supplementary Fig. 9 and 10), or if Cact levels are changed by 2 orders of magnitude to simulate the change due to Toll signaling (Supplementary Fig. 11–13). Since we calculate expression relative to WT, the results depend to a lesser extent on the absolute values of the parameters (Supplementary SI-2), which are taken from previous studies. In this simplified model, the assumption of steady state disallows the possibility of simulating the time-dependent response to a change in stimulus. Therefore, the calculated values should be regarded as qualitative trends. Nevertheless, the simulation results suggest the necessary (though not sufficient) step among all those considered, and indicates that SUMOylation is likely to be associated with a lower transcriptional ability. Since DL^U^ seems to be a better transcriptional activator, SUMOylation of DL may be a general mechanism to reduce occupancy of DL^U^ at the promoter regions. Our mathematical model suggests that DL^S^ dimers bind to the promoter and block access to the more transcriptionally efficient DL^U^ dimers, thus attenuating transcription. Additionally, DL^S^ may be deficient or less efficient in its ability to interact with the core transcriptional machinery or with partner basic helix–loop–helix proteins. DL^S^ is, in all probability, a nonfunctional variant of DL. Alternatively, though not directly supported by our data, is the possibility that DL^S^, when bound to DNA can recruit a repressor and subsequently lead to deacetylation of the chromatin that is resistant to transcription. SUMO-mediated attenuation of DL activity thus adds another layer to the complex regulation of Toll/NF-κB signaling.

## Data availability

Strains and plasmids are available upon request. The authors affirm that all data necessary for confirming the conclusions of the article are present within the article, figures, tables, and Supplementary material.

*Supplemental material* is available at *GENETICS* online.

## Supplementary Material

iyac081_Supplemental_Figures

iyac081_Supplemental_SI-2

iyac081_Supplemental_Material_Table_1

iyac081_Supplemental_Material_SI

iyac081_Supplemental_Material_Legends
