# Supplementary material for: SUMOylation of Dorsal attenuates Toll/NF-κB signaling
Source: Genetics. 2022 May 14;221(3):iyac081. doi: 10.1093/genetics/iyac081 (PMC9252280; doi:10.1093/genetics/iyac081)
Supplement: iyac081_Supplemental_Figures [file iyac081_supplemental_figures.pdf]

Fig. S1

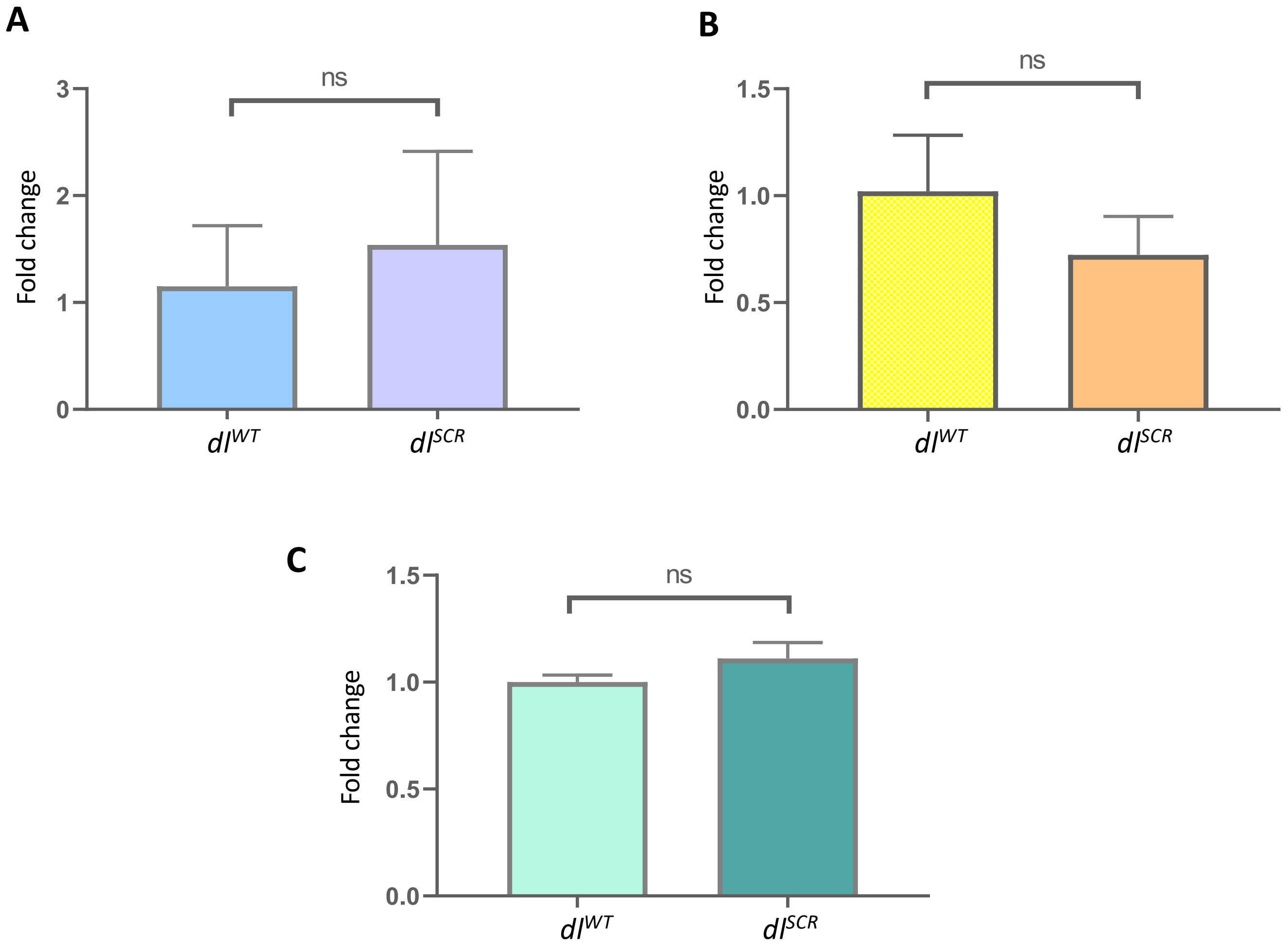

Fig. S1. *dl* transcripts express at similar levels in *dl*^{WT} and *dl*^{SCR}. *dl* transcript levels assayed by qRT-PCR across different stages of the fly life cycle- embryo (A), larva (B) and adult (D) for the genotypes *dl*^{WT} and *dl*^{SCR}.

Fig. S2

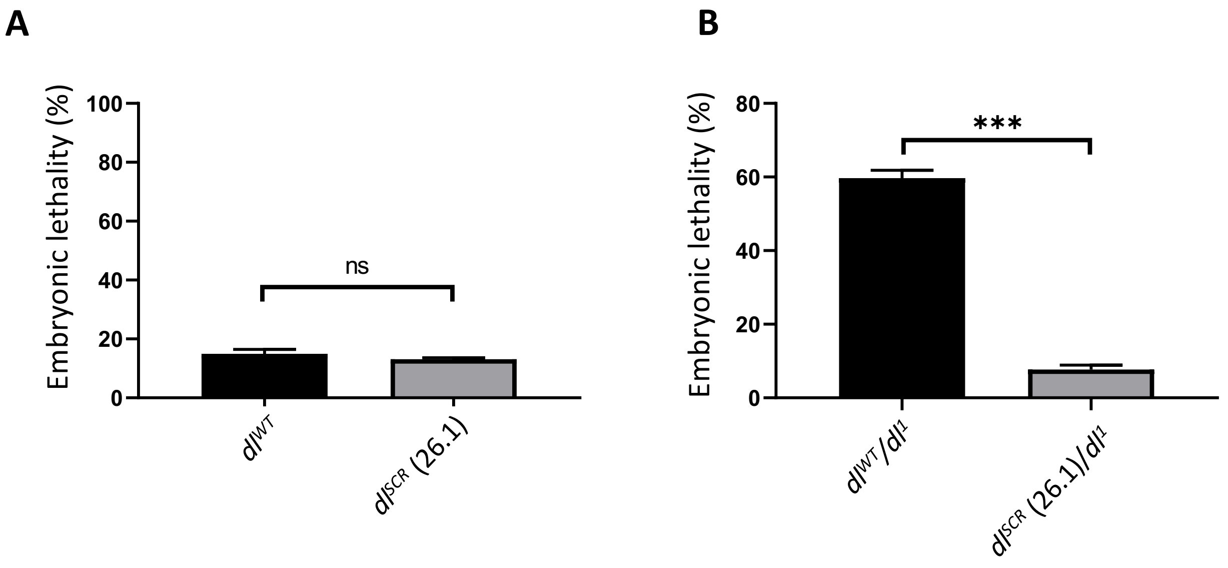

Fig. S2. Haplo-sufficiency of the *dl*^{SCR} allele. Embryonic lethality is plotted for the indicated genotypes, at 29 °C. N=3, mean ± SEM, Unpaired t-test, (ns) P >0.05, (***) P < 0.001. Embryonic hatching suggests viability.

Fig. S3

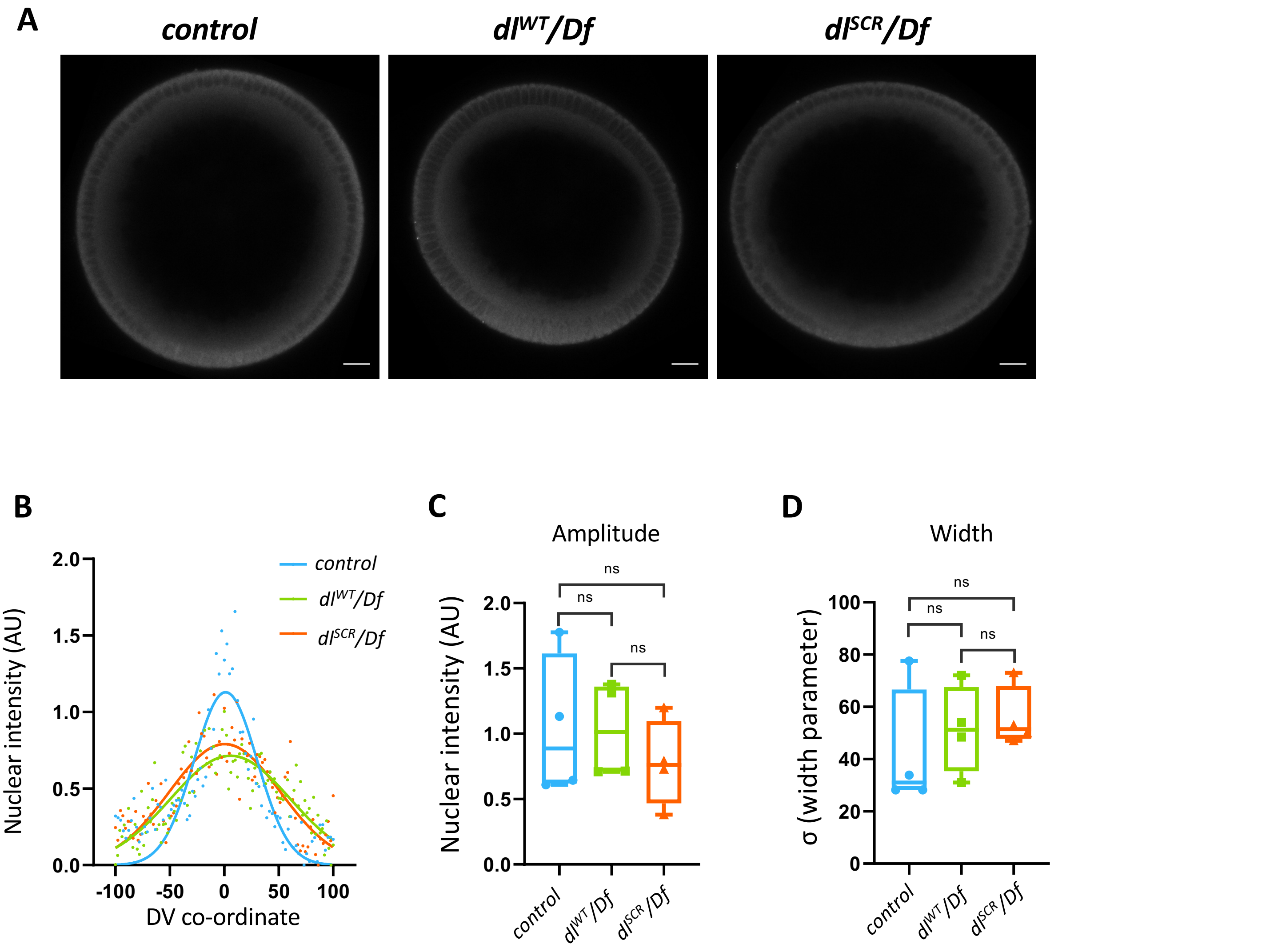

Fig. S3. **The DL gradient visualized with a DL antibody in *control*, dl^{WT}/Df, and dl^{SCR}/Df embryos** (A) Transverse sections of nc 14 embryos of the specified genotypes. (B) shows representative intensity profiles of *control*, dl^{WT}/Df and dl^{SCR}/Df embryos, fitted to a Gaussian. The amplitude (C) and width (D) of the gradient centered at the ventral midline is plotted. n=4, ordinary one-way ANOVA, (ns) P >0.05.

Fig. S4

A

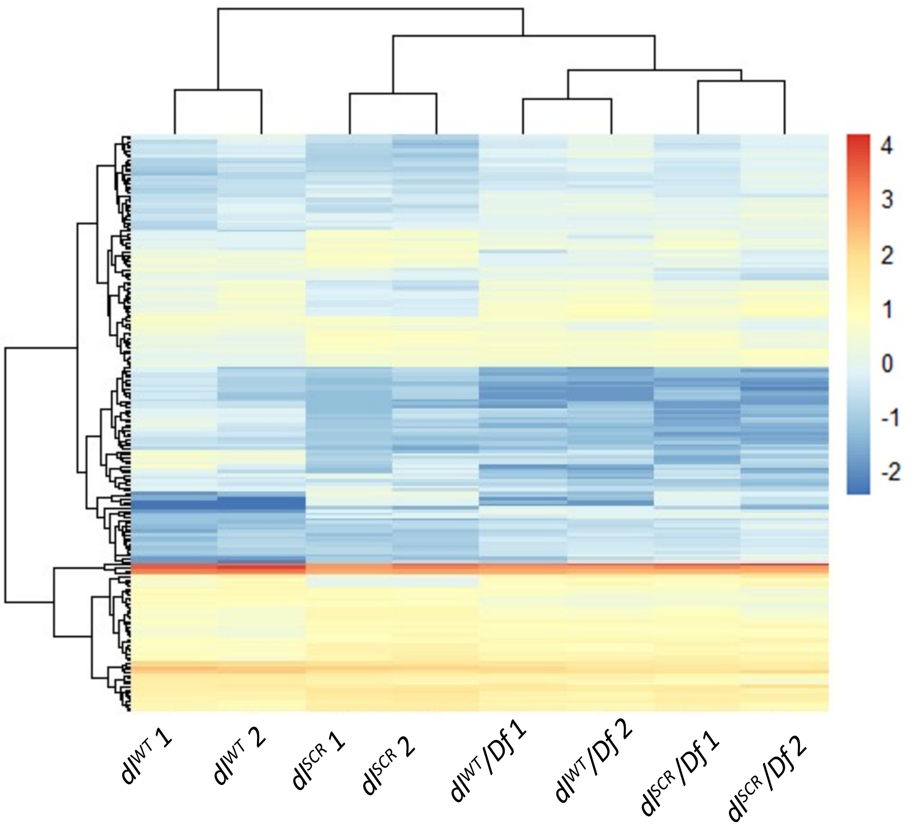

C

Gene	log2 fold change	Gene network
zen	1.64	D-V
kni	1.34	A-P
twi	1.33	D-V
rho	1.32	D-V
sna	1.08	D-V
ftz	1.06	A-P
oc	1.04	A-P
Egfr	0.99	EGF
tll	0.985	A-P
opa	0.982	A-P
tld	0.92	D-V
run	0.91	D-V
ths	0.86	D-V
hkb	0.85	A-P

B

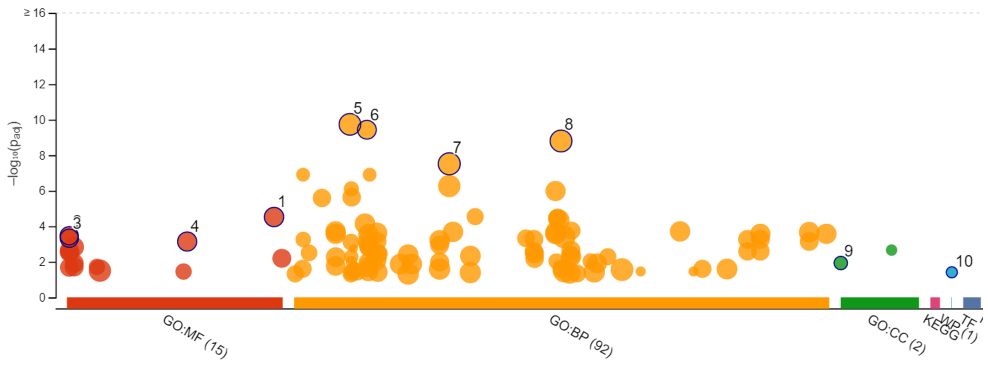

GO term

1. transcription regulator activity
2. RNA polymerase II cis-regulatory region sequence-specific DNA binding
3. cis-regulatory region sequence-specific DNA binding
4. sequence-specific DNA binding
5. multicellular organism development
6. embryo development
7. developmental process
8. anatomical structure development
9. histone deacetylase complex
10. Hedgehog Signaling Pathway

Fig. S4. **Quantitative changes in the transcriptome of embryos laid by *dl*^{SCR} mothers** (A) Heat map of differentially expressed genes across *dl*^{WT}, *dl*^{SCR}, *dl*^{WT}*/Df*, *dl*^{SCR}*/Df are* represented, in duplicates. Scaled, LogCPM values are plotted. (B) denotes significantly enriched gene ontology (GO) terms, for genes in (A), categorized by molecular function (MF), biological process (BP), cellular component (CC) and pathway (KEGG). A subset of differentially regulated genes compared across *dl*^{WT}*/Df* and *dl*^{SCR}*/Df* with known binding sites for DL is listed in (C), along with the corresponding log2 fold-change values. The genes are broadly classified according to the gene regulatory network they are a part of in the early embryo: D-V (Dorso-ventral patterning; in purple), A-P (Antero-posterior patterning; in pink) and the EGF signaling pathway (in blue).

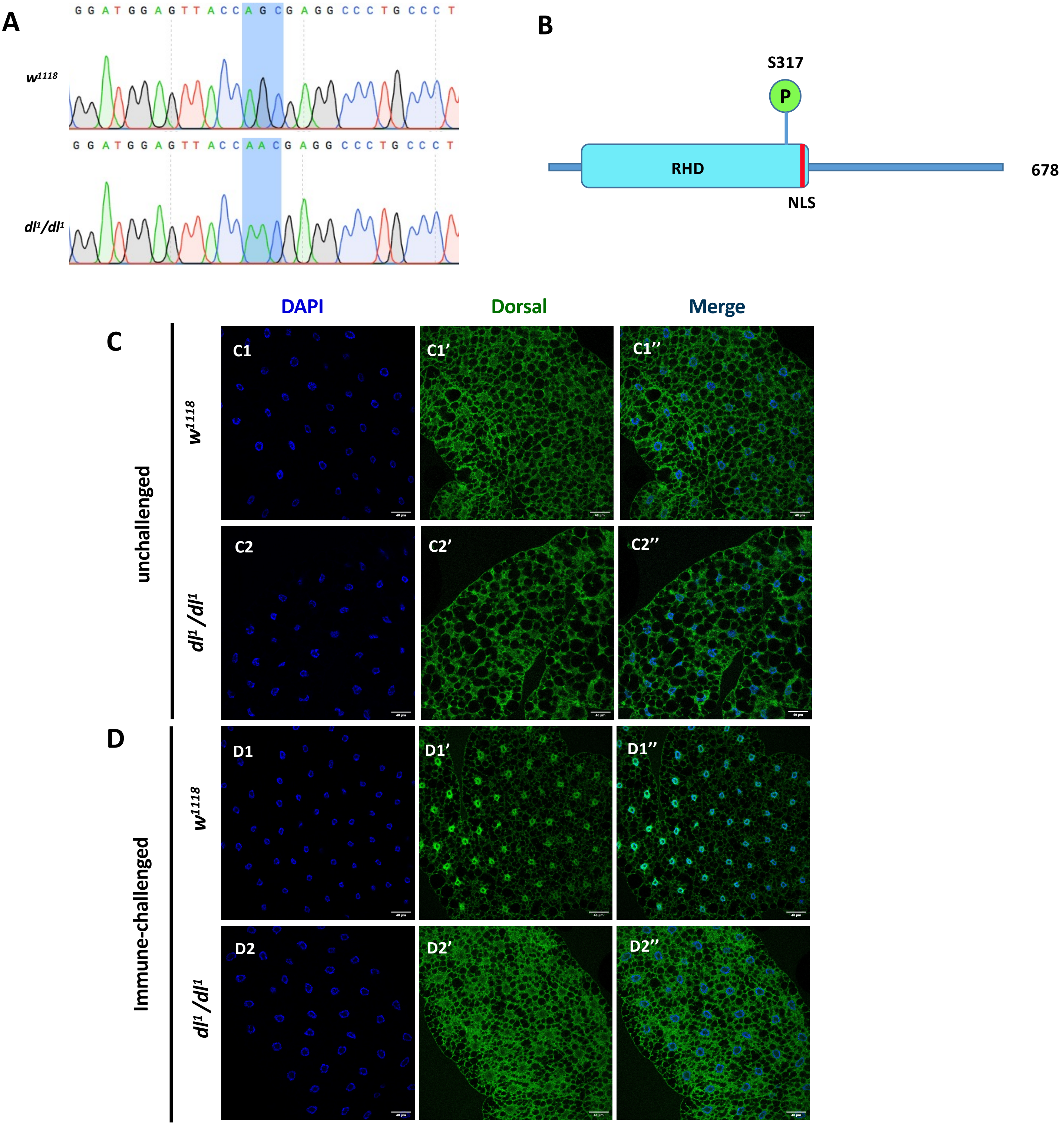

Fig. S5. *dl*^{1} is a S317N mutant and is resistant to Toll signalling. The CDS of *dl*^{1}/*dl*^{1} was sequenced and found to harbour a mutation in a critical S317 residue in the rel-homology domain, subject to a phospho-modification (A). The presence of the S317N mutation is highlighted in blue, in (B), along with a reference sequence for *w*^{1118}. In the unchallenged condition, immunostaining with a DL antibody reveals a pre-dominantly cytoplasmic distribution of DL in *dl*^{1}/*dl*^{1} and *w*^{1118} (C'-C'') in the fat body. Septic injury with *S. saprophyticus* triggers the nuclear migration of DL in the wild-type animal (D1-D1''), while DL1 is retained in the cytoplasm (D2-D2''). Nuclei are marked with DAPI. N=3, n=5

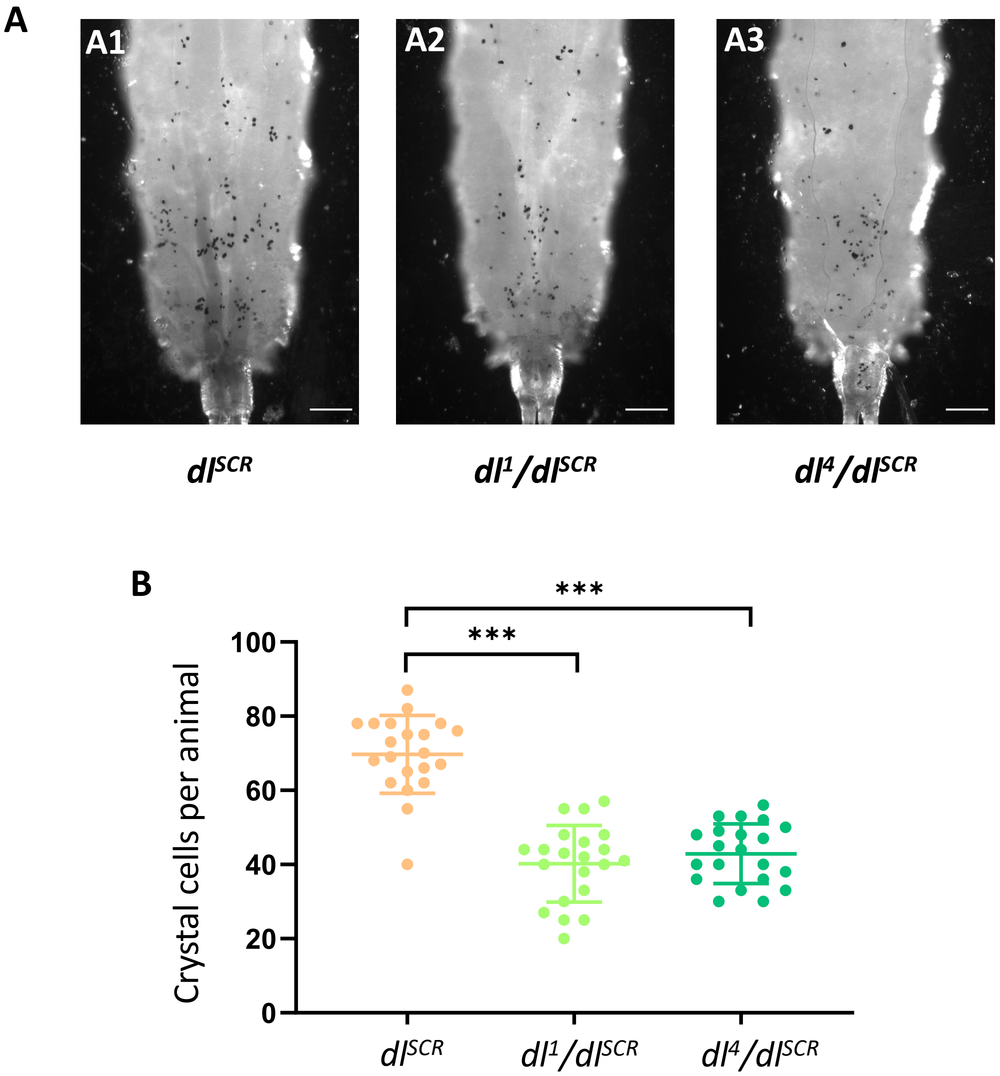

Fig. S6. **Crystal cells in *dl^SCR^*, *dl^1^/dl^SCR^* , *and dl^4^/dl^SCR^* larvae***.* Crystal cells in the third-instar larva were observed under a bright-field microscope, for the genotypes indicated in (A). The last three posterior segments were imaged with the dorsal side facing the viewer. The number of crystal cells in the posterior segments were counted and are plotted in (B). n=20, Mean ± SEM, ordinary one-way ANOVA, (***) $P < 0.0001$

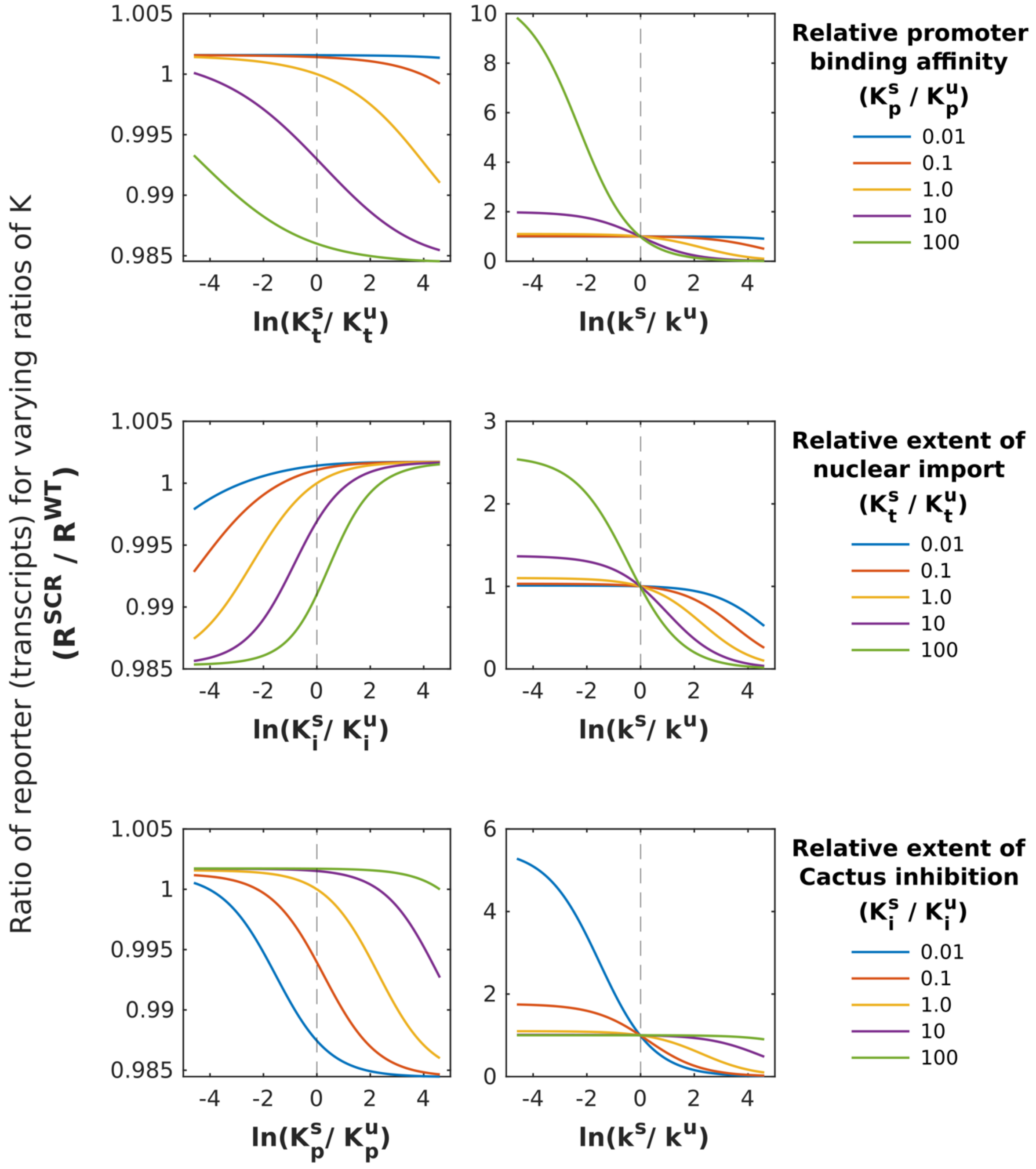

Fig. S7. Simulating the effect of DL SUMOylation: The Simulation in Fig 8B is repeated by varying different combinations of parameters, including the the k^s/k^u and K_p^s/K_p^u combination (top right panel) which is repeated from Fig 8B. The reporter expression level for the DL^{SCR} is unchanged from the corresponding WT level when k^s/k^u is one, *i.e.* the relative expression efficiency is unchanged due to SUMOylation, despite 100-fold changes in combinations of two parameters (left column all rows). However, decrease in k^s/k^u together with a change that results in higher nuclear DL^S due to lesser Cact inhibition (bottom right) or higher partitioning (middle right); or tighter promoter binding of dimers containing DL^S (top right) result in higher expression in the DL^{SCR} relative to the corresponding WT level. Other parameter values are taken from SI-2.

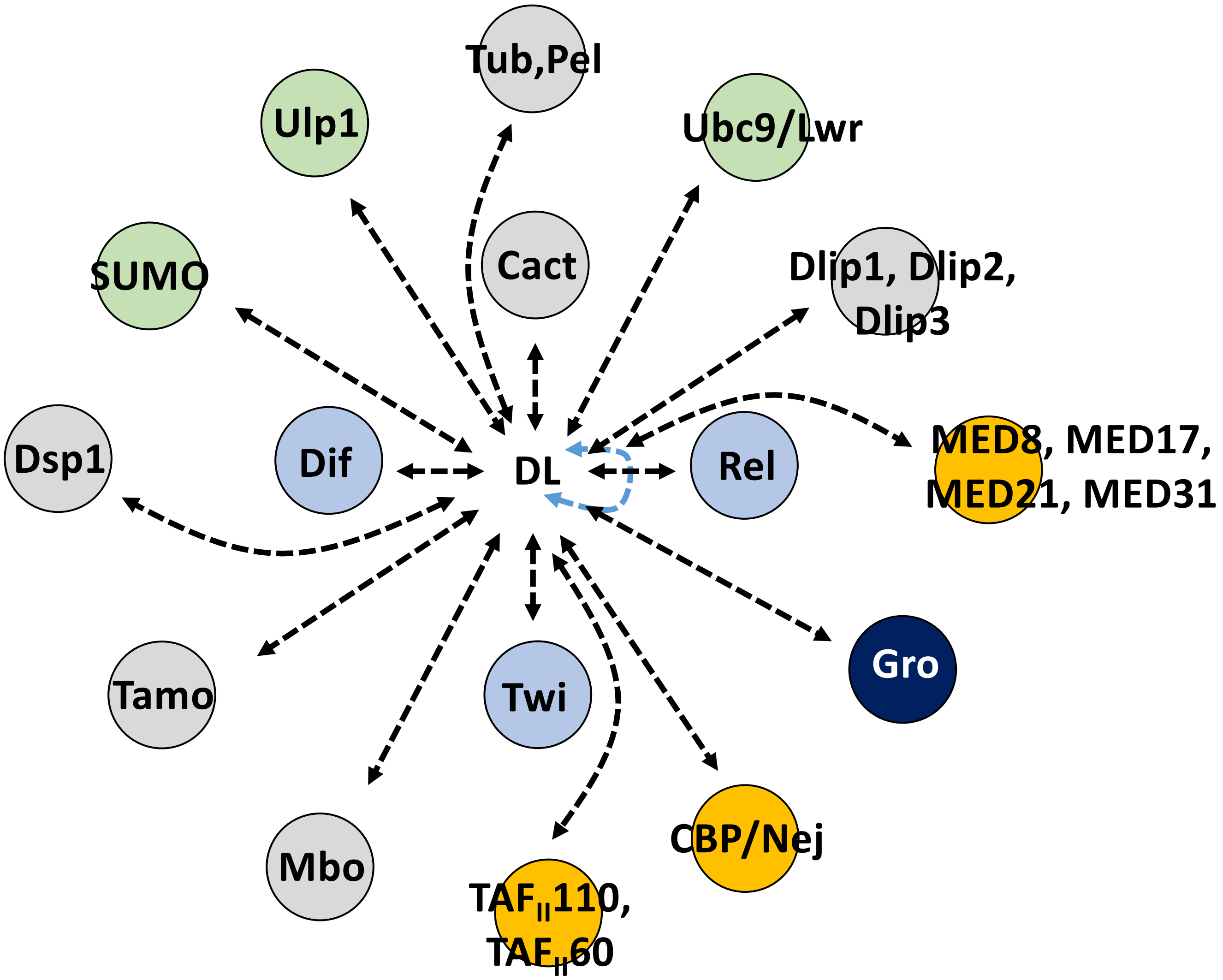

Fig. S8. Summary of known physical interactors of DL. A list of DL interactors based on data collated in Flybase. The SUMO conjugation and de-conjugation machinery (green circles) interacts with DL, as does the machinery involved in transcriptional activation (orange circles). SUMOylation of DL may perturb the interaction of DL with any-one or all of these partner proteins and in turn may influence DL mediated activation or repression.

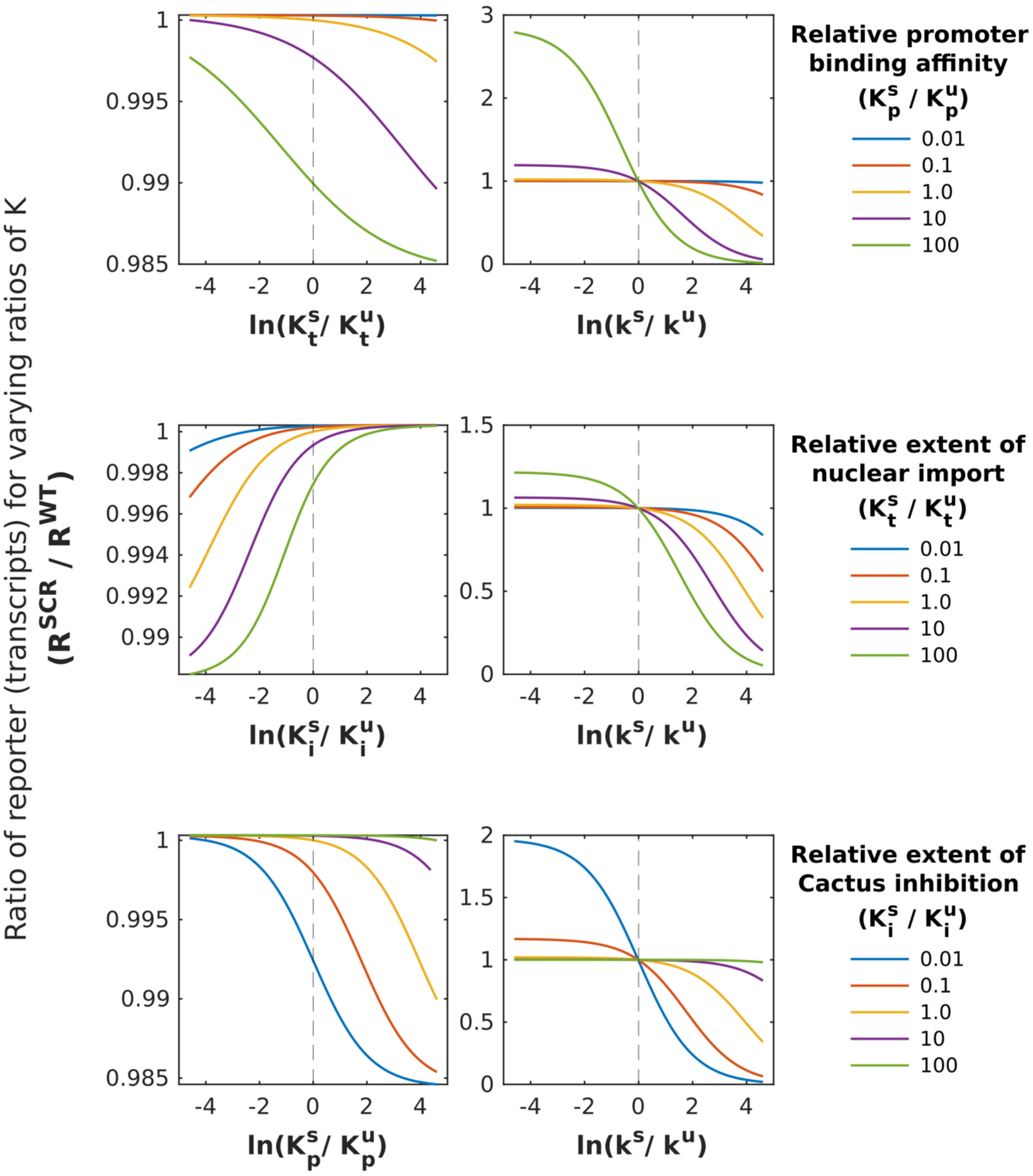

Fig. S9. Simulating the effect of a lower fraction of SUMOylated DL. Relative expression ratio as a function of changes in parameter combinations attributed to SUMOylated DL. Parameters and simulations are identical to Fig. S7, except that total SUMOylated DL in WT was assumed to be 1% of the total DL in the system.

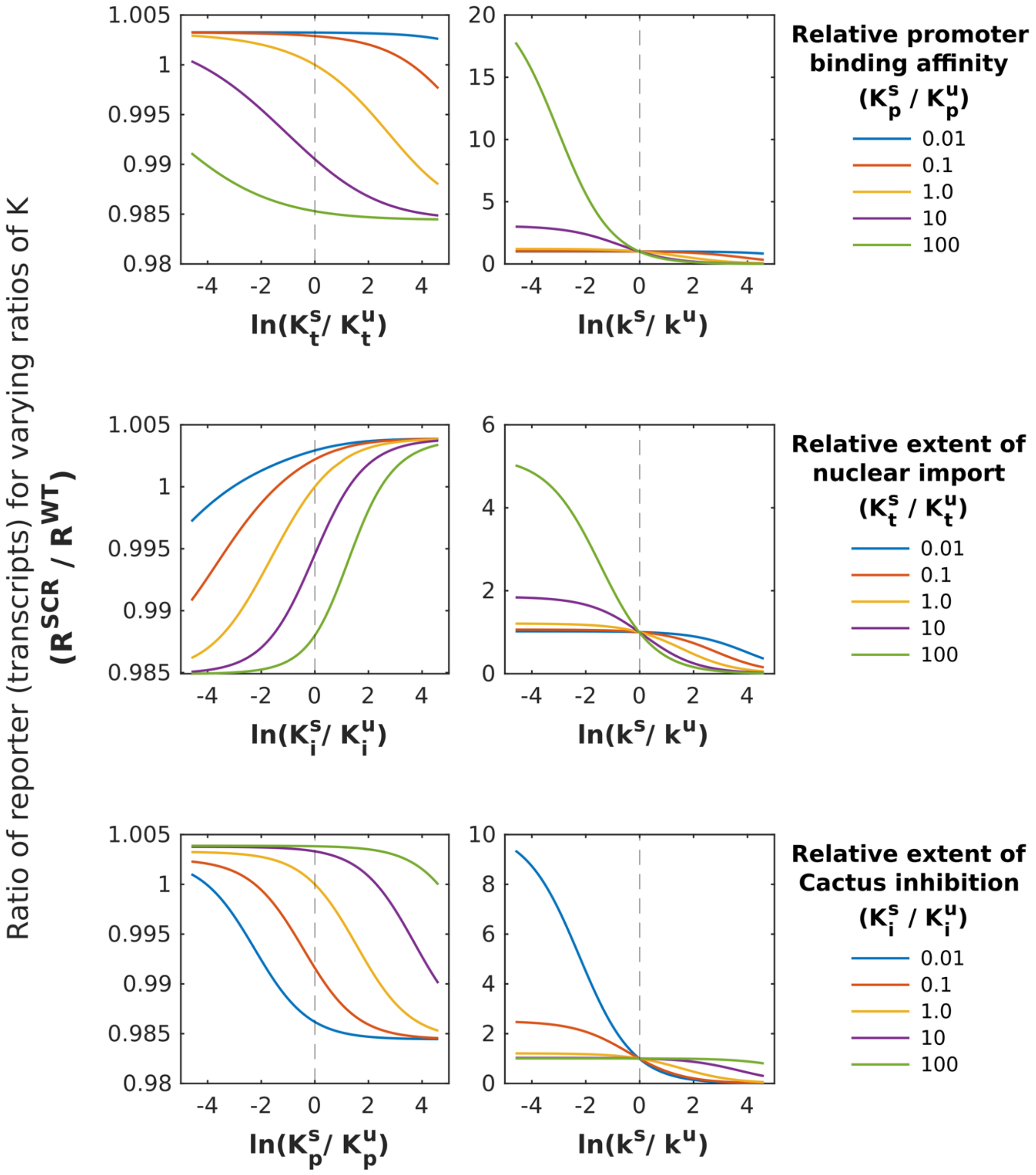

Fig. S10. Simulating the effect of a higher fraction of SUMOylated DL. Relative expression ratio as a function of changes in parameter combinations attributed to SUMOylated DL. Parameters and simulations are identical to Fig. S7, except that total SUMOylated DL in WT was assumed to be 10% of the total DL in the system.

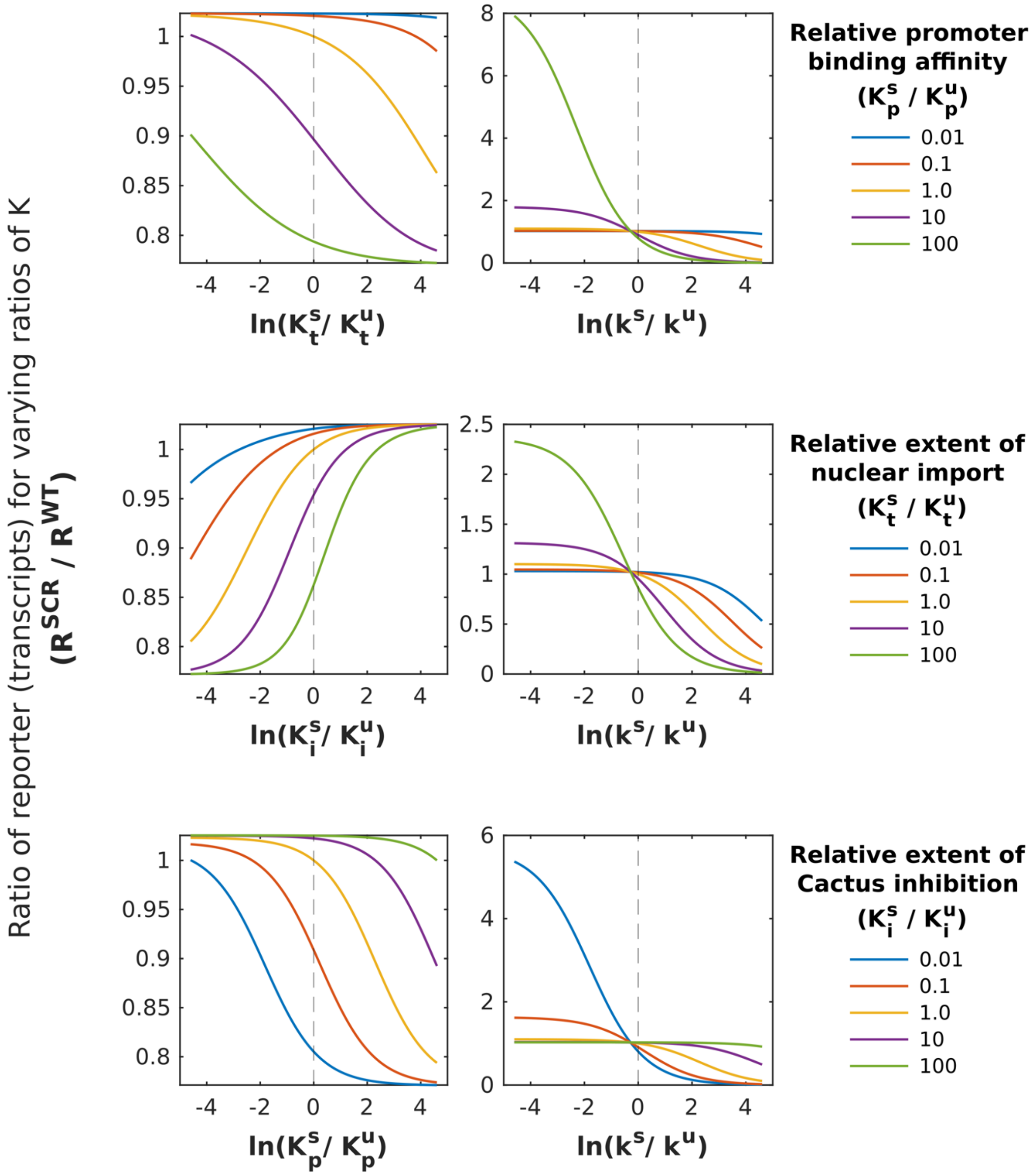

Fig. S11. Simulating the effect of increase in cytosolic Cact levels. Relative expression ratio as a function of changes in parameter combinations attributed to SUMOylated DL. Parameters and simulations are identical to Fig. S7, except that total Cact in both WT and mutant was increased by 10-fold (Total Cact $= 10^6$ molecules).

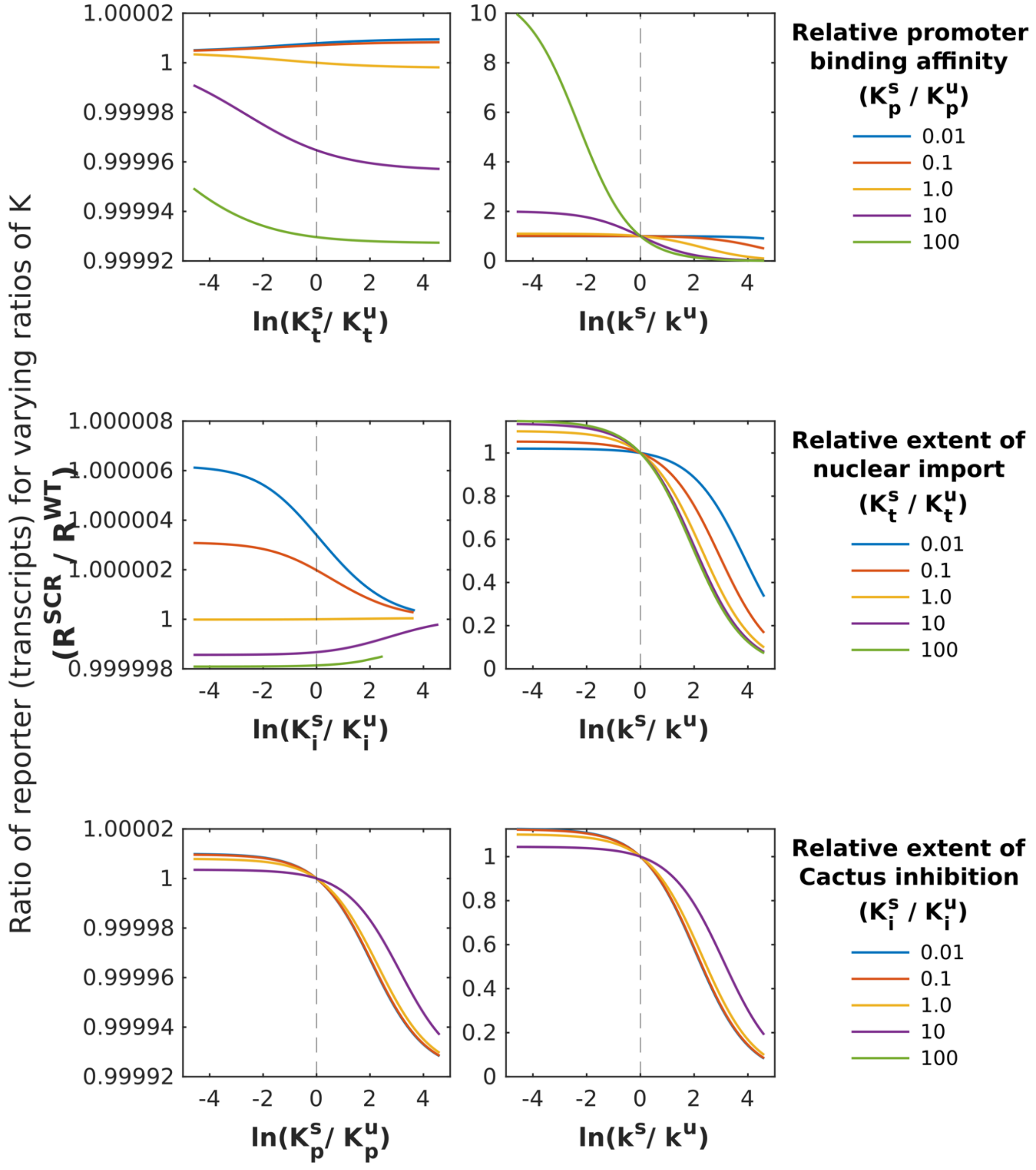

Fig. S12. Simulating the effect of 10X lower cytosolic Cact levels. Relative expression ratio as a function of changes in parameter combinations attributed to SUMOylated DL. Parameters and simulations are identical to Fig. S7, except that total Cact in both WT and mutant was decreased by 10-fold (Total Cact = 10^4 molecules).

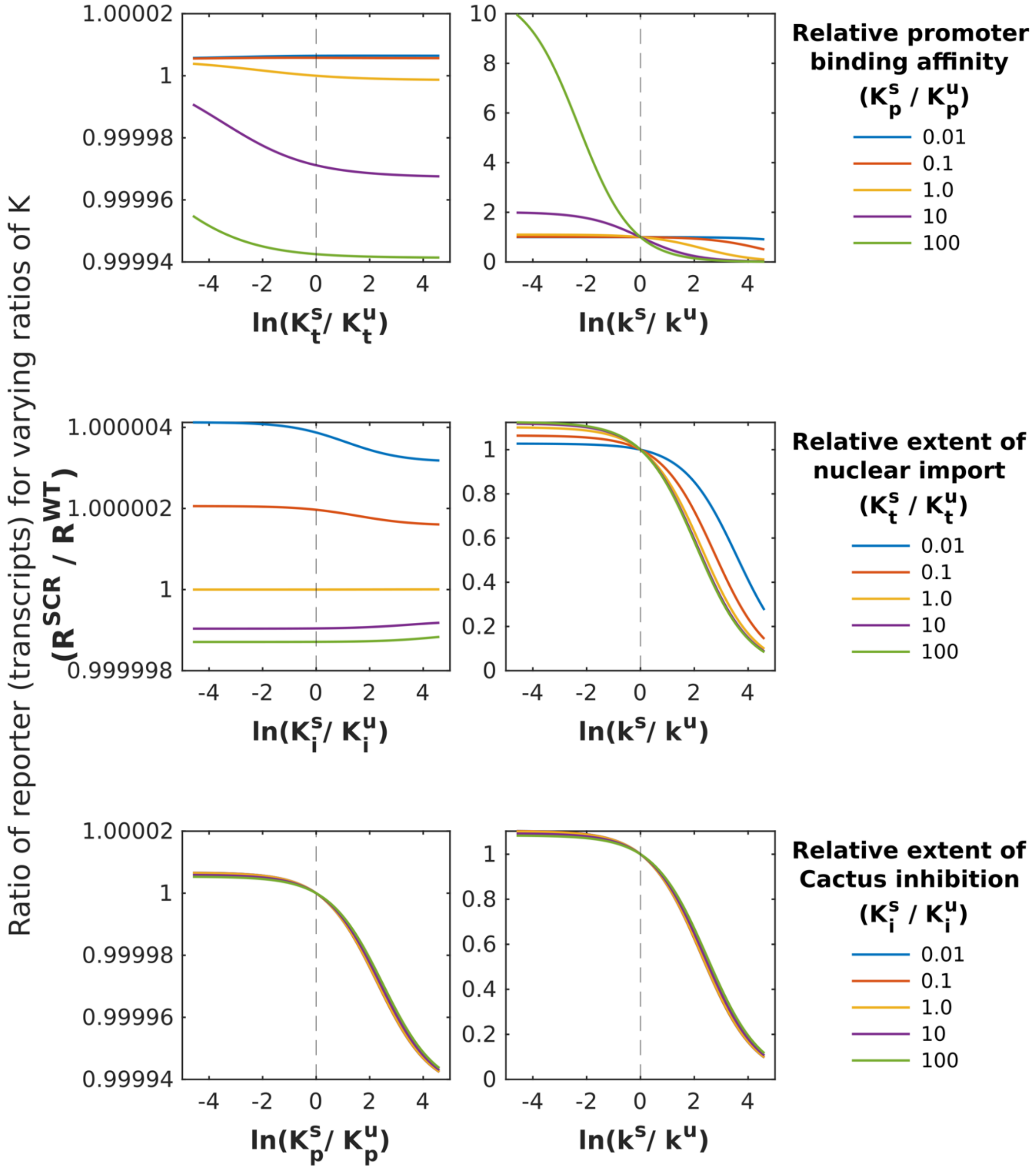

Fig. S13. Simulating the effect of 100X lower cytosolic Cact levels. Relative expression ratio as a function of changes in parameter combinations attributed to SUMOylated DL. Parameters and simulations are identical to Fig. S7, except that total Cact in both WT and mutant was decreased by 100-fold (Total Cact = 10^3 molecules).
