# Supplementary material for: SUMOylation of Dorsal attenuates Toll/NF-κB signaling
Source: Genetics. 2022 May 14;221(3):iyac081. doi: 10.1093/genetics/iyac081 (PMC9252280; doi:10.1093/genetics/iyac081)
Supplement: iyac081_Supplemental_SI-2 [file iyac081_supplemental_si-2.docx]

# SI-2: Parameter Table. Parameter values used for simulations are summarized in the following table. Parameter values for SUMOylated homodimer and heterodimers are initially kept same as the values shown below, and were then varied as explained in the Methods section.

| **Model parameter** | **Value** | **Reference** |
| --- | --- | --- |
| Total number of DL monomers (${DL}_{Total}^{u}+{DL}_{Total}^{s}$) | ${10}^{5}$ | *Tay et al. 2010* |
| Number of Cact molecules (${Cact}_{Total}$) | ${10}^{5}$^†^ | *Kanodia et al. 2009,  Tay et al. 2010* |
| Number of promoter sites ($P_{Total}$) | ${10}^{2}$ | *Assumed* |
| DL dimerization | $K_{D}=\frac{k_{D+}}{k_{D-}}=4.06\times{10}^{4}$ | *Ramsey et al. 2019^‡^* |
| Cact inhibition of DL dimers | $K_{i}=\frac{k_{i+}}{k_{i-}}=0.025$ | *Tay et al. 2010* |
| Transport of DL dimers into nucleus | $K_{t}=\frac{k_{t+}}{k_{t-}}=4$ | *Carrell et al. 2017,  Al Asafen et al. 2020* |
| DL dimer binding to promoter region | $K_{p}=\frac{k_{p+}}{k_{p-}}=0.4$ | *Tay et al. 2010* |
| Rate of reporter mRNA synthesis | $k=0.5 s^{-1}$ | *Tay et al. 2010* |

^†^ The number of Cact molecules is not available. Approximate computations using the formation, degradation rates show that number of Cact molecules is ~ ${10}^{5}$. Kanodia et al. (2009) suggest that amounts of DL and Cact in the system should be comparable.

^‡^ Approximated assuming a cell volume of 1.5 pL.

# References

1. Tay, S. et al. Single-cell NF-κB dynamics reveal digital activation and analogue information processing. *Nature* vol. 466 267–271 (2010).
2. Kanodia, J. S. et al. Dynamics of the Dorsal morphogen gradient. *Proceedings of the National Academy of Sciences* vol. 106 21707–21712 (2009).
3. Ramsey, K. M., Chen, W., Marion, J. D., Bergqvist, S. & Komives, E. A. *Exclusivity and Compensation in NFκB Dimer Distributions and IκB Inhibition*. Biochemistry vol. 58 2555–2563 (2019).
4. Carrell, S. N. et al. A facilitated diffusion mechanism establishes the Drosophila Dorsal gradient. *Development* (2017) doi:10.1242/dev.155549.
5. Al Asafen, H. et al. Robustness of the Dorsal morphogen gradient with respect to morphogen dosage. *PLOS Computational Biology* vol. 16 e1007750 (2020).
