# Supplementary material for: SUMOylation of Dorsal attenuates Toll/NF-κB signaling
Source: Genetics. 2022 May 14;221(3):iyac081. doi: 10.1093/genetics/iyac081 (PMC9252280; doi:10.1093/genetics/iyac081)
Supplement: iyac081_Supplemental_Material_SI [file iyac081_supplemental_material_si.docx]

# SI-1: Mathematical model. The effect of DL on reporter gene expression was modelled as described in the Materials and Methods section (depicted in Fig. 8A). SUMOylated DL dimers ($\mathbf{DL}^{\boldsymbol{u}}\boldsymbol{.}\mathbf{DL}^{\boldsymbol{s}}$ and $\mathbf{DL}^{\boldsymbol{s}}\boldsymbol{.}\mathbf{DL}^{\boldsymbol{s}}$) are assumed to participate in all reactions but with different specific rate constants relative to the unSUMOylated dimer,$\mathbf{DL}^{\boldsymbol{u}}\boldsymbol{:}\mathbf{DL}^{\boldsymbol{u}}$. Individual reactions shown in Fig. 8A can be summarized as follows.

*Wild Type:*

| Dimerization:  $K_{D}^{u} : 2 \mathrm{DL}_{c/n}^{u} \leftrightarrow\mathrm{DL}^{u}.\mathrm{DL}_{c/n}^{u}$ $K_{D}^{s} : 2 \mathrm{DL}_{c/n}^{s} \leftrightarrow\mathrm{DL}^{s}.\mathrm{DL}_{c/n}^{s}$ $K_{D}^{us} : \mathrm{DL}_{c/n}^{s}+\mathrm{DL}_{c/n}^{u} \leftrightarrow\mathrm{DL}^{u}.\mathrm{DL}_{c/n}^{s}$ | Inhibition by Cactus:  $K_{i}^{u} : \mathrm{DL}^{u}.\mathrm{DL}_{c}^{u}+\mathrm{Cact}\leftrightarrow\mathrm{DL}^{u}.\mathrm{DL}^{u}\text{–}\mathrm{Cact}$ $K_{i}^{s} : \mathrm{DL}^{s}.\mathrm{DL}_{c}^{s}+\mathrm{Cact}\leftrightarrow\mathrm{DL}^{s}.\mathrm{DL}^{s}\text{–}\mathrm{Cact}$ $K_{i}^{us} : \mathrm{DL}^{u}.\mathrm{DL}_{c}^{s}+\mathrm{Cact}\leftrightarrow\mathrm{DL}^{u}.\mathrm{DL}^{s}\text{–}\mathrm{Cact}$ |
| --- | --- |
| Transport into nucleus:  $K_{t}^{u} : \mathrm{DL}^{u}.\mathrm{DL}_{c}^{u} \leftrightarrow\mathrm{DL}^{u}.\mathrm{DL}_{n}^{u}$ $K_{t}^{s} : \mathrm{DL}^{s}.\mathrm{DL}_{c}^{s} \leftrightarrow\mathrm{DL}^{s}.\mathrm{DL}_{n}^{s}$ $K_{t}^{us} : \mathrm{DL}^{u}.\mathrm{DL}_{c}^{s} \leftrightarrow\mathrm{DL}^{u}.\mathrm{DL}_{n}^{s}$ | Binding to promoter region:  $K_{p}^{u} : \mathrm{DL}^{u}.\mathrm{DL}_{n}^{u}+P \leftrightarrow\mathrm{DL}^{u}.\mathrm{DL}^{u}\text{–}P$ $K_{p}^{s} : \mathrm{DL}^{s}.\mathrm{DL}_{n}^{s}+P \leftrightarrow\mathrm{DL}^{s}.\mathrm{DL}^{s}\text{–}P$ $K_{p}^{us} : \mathrm{DL}^{u}.\mathrm{DL}_{n}^{s}+P \leftrightarrow\mathrm{DL}^{u}.\mathrm{DL}^{s}\text{–}P$ |

*SCR mutant:*

| Dimerization:  $K_{D}^{u} : 2 \mathrm{DL}_{c/n}^{u} \leftrightarrow\mathrm{DL}^{u}.\mathrm{DL}_{c/n}^{u}$ | Inhibition by Cactus:  $K_{i}^{u} : \mathrm{DL}^{u}.\mathrm{DL}_{c}^{u}+\mathrm{Cact}\leftrightarrow\mathrm{DL}^{u}.\mathrm{DL}^{u}\text{–}\mathrm{Cact}$ |
| --- | --- |
| Transport into nucleus:  $K_{t}^{u} : \mathrm{DL}^{u}.\mathrm{DL}_{c}^{u} \leftrightarrow\mathrm{DL}^{u}.\mathrm{DL}_{n}^{u}$ | Binding to promoter region:  $K_{p}^{u} : \mathrm{DL}^{u}.\mathrm{DL}_{n}^{u}+P \leftrightarrow\mathrm{DL}^{u}.\mathrm{DL}^{u}\text{–}P$ |

Here, superscripts $u, s$ represent the SUMOylation status and subscripts $n, c$ denote the nuclear, cytosolic compartments, respectively. Uppercase $K$ in each reaction correspond to the ratio of forward to backward reaction rate constants. That is, $K_{\varepsilon}^{u}={k_{\varepsilon+}^{u}}/{k_{\varepsilon-}^{u}}$,$K_{\varepsilon}^{s}={k_{\varepsilon+}^{s}}/{k_{\varepsilon-}^{s}}$ and $K_{\varepsilon}^{us}={k_{\varepsilon+}^{us}}/{k_{\varepsilon-}^{us}}$, where $\varepsilon=D,i,t,p$. It is assumed that in the time interval being considered, total protein amounts are conserved and free and bound protein concentrations are at a (pseudo) steady state. A mass balance on individual proteins and complexes results in the following set of equations (Eq. 1-22).

| $0={d\left[ \mathrm{DL}_{c}^{u} \right]}/{dt}=-2k_{D+}^{u}\left[ \mathrm{DL}_{c}^{u} \right]^{2}+2k_{D-}^{u}\left[ \mathrm{DL}^{u}.\mathrm{DL}_{c}^{u} \right]-k_{D+}^{us}\left[ \mathrm{DL}_{c}^{u} \right]\left[ \mathrm{DL}_{c}^{s} \right]+k_{D-}^{us}\left[ \mathrm{DL}^{u}.\mathrm{DL}_{c}^{s} \right]$ | Eq. 1 |
| --- | --- |
| $0={d\left[ \mathrm{DL}_{c}^{s} \right]}/{dt}=-2k_{D+}^{s}\left[ \mathrm{DL}_{c}^{s} \right]^{2}+2k_{D-}^{s}\left[ \mathrm{DL}^{s}.\mathrm{DL}_{c}^{s} \right]-k_{D+}^{us}\left[ \mathrm{DL}_{c}^{u} \right]\left[ \mathrm{DL}_{c}^{s} \right]+k_{D-}^{us}\left[ \mathrm{DL}^{u}.\mathrm{DL}_{c}^{s} \right]$ | Eq. 2 |
| $0={d\left[ \mathrm{DL}^{u}.\mathrm{DL}_{c}^{u} \right]}/{dt}=-k_{D-}^{u}\left[ \mathrm{DL}^{u}.\mathrm{DL}_{c}^{u} \right]+k_{D+}^{u}\left[ \mathrm{DL}_{c}^{u} \right]^{2}-k_{i+}^{u}\left[ \mathrm{DL}^{u}.\mathrm{DL}_{c}^{u} \right]\left[ \mathrm{Cact} \right]$ $+k_{i-}^{u}\left[ \mathrm{DL}^{u}.\mathrm{DL}^{u}\text{–}\mathrm{Cact} \right]-k_{t+}^{u}\left[ \mathrm{DL}^{u}.\mathrm{DL}_{c}^{u} \right]+k_{t-}^{u}\left[ \mathrm{DL}^{u}.\mathrm{DL}_{n}^{u} \right]$ | Eq. 3 |
| $0={d\left[ \mathrm{DL}^{s}.\mathrm{DL}_{c}^{s} \right]}/{dt}=-k_{D-}^{s}\left[ \mathrm{DL}^{s}.\mathrm{DL}_{c}^{s} \right]+k_{D+}^{s}\left[ \mathrm{DL}_{c}^{s} \right]^{2}-k_{i+}^{s}\left[ \mathrm{DL}^{s}.\mathrm{DL}_{c}^{s} \right]\left[ \mathrm{Cact} \right]$ $+k_{i-}^{s}\left[ \mathrm{DL}^{s}.\mathrm{DL}^{s}\text{–}\mathrm{Cact} \right]-k_{t+}^{s}\left[ \mathrm{DL}^{s}.\mathrm{DL}_{c}^{s} \right]+k_{t-}^{s}\left[ \mathrm{DL}^{s}.\mathrm{DL}_{n}^{s} \right]$ | Eq. 4 |
| $0={d\left[ \mathrm{DL}^{u}.\mathrm{DL}_{c}^{s} \right]}/{dt}=-k_{D-}^{us}\left[ \mathrm{DL}^{u}.\mathrm{DL}_{c}^{s} \right]+k_{D+}^{us}\left[ \mathrm{DL}_{c}^{u} \right]\left[ \mathrm{DL}_{c}^{s} \right]-k_{i+}^{us}\left[ \mathrm{DL}^{u}.\mathrm{DL}_{c}^{s} \right]\left[ \mathrm{Cact} \right]$ $+k_{i-}^{us}\left[ \mathrm{DL}^{u}.\mathrm{DL}^{s}\text{–}\mathrm{Cact} \right]-k_{t+}^{us}\left[ \mathrm{DL}^{u}.\mathrm{DL}_{c}^{s} \right]+k_{t-}^{us}\left[ \mathrm{DL}^{u}.\mathrm{DL}_{n}^{s} \right]$ | Eq. 5 |
| $0={d\left[ \mathrm{DL}^{u}.\mathrm{DL}^{u}\text{–}\mathrm{Cact} \right]}/{dt}=k_{i+}^{u}\left[ \mathrm{DL}^{u}.\mathrm{DL}_{c}^{u} \right]\left[ \mathrm{Cact} \right]-k_{i-}^{u}\left[ \mathrm{DL}^{u}.\mathrm{DL}^{u}\text{–}\mathrm{Cact} \right]$ | Eq. 6 |
| $0={d\left[ \mathrm{DL}^{s}.\mathrm{DL}^{s}\text{–}\mathrm{Cact} \right]}/{dt}=k_{i+}^{s}\left[ \mathrm{DL}^{s}.\mathrm{DL}_{c}^{s} \right]\left[ \mathrm{Cact} \right]-k_{i-}^{s}\left[ \mathrm{DL}^{s}.\mathrm{DL}^{s}\text{–}\mathrm{Cact} \right]$ | Eq. 7 |
| $0={d\left[ \mathrm{DL}^{u}.\mathrm{DL}^{s}\text{–}\mathrm{Cact} \right]}/{dt}=k_{i+}^{us}\left[ \mathrm{DL}^{u}.\mathrm{DL}_{c}^{s} \right]\left[ \mathrm{Cact} \right]-k_{i-}^{us}\left[ \mathrm{DL}^{u}.\mathrm{DL}^{s}\text{–}\mathrm{Cact} \right]$ | Eq. 8 |
| $0={d\left[ \mathrm{Cact} \right]}/{dt}=-k_{i+}^{us}\left[ \mathrm{DL}^{u}.\mathrm{DL}_{c}^{s} \right]\left[ \mathrm{Cact} \right]+k_{i-}^{us}\left[ \mathrm{DL}^{u}.\mathrm{DL}^{s}\text{–}\mathrm{Cact} \right]-k_{i+}^{u}\left[ \mathrm{DL}^{u}.\mathrm{DL}_{c}^{u} \right]\left[ \mathrm{Cact} \right]$ $+k_{i-}^{u}\left[ \mathrm{DL}^{u}.\mathrm{DL}^{u}\text{–}\mathrm{Cact} \right]-k_{i+}^{s}\left[ \mathrm{DL}^{s}.\mathrm{DL}_{c}^{s} \right]\left[ \mathrm{Cact} \right]+k_{i-}^{s}\left[ \mathrm{DL}^{s}.\mathrm{DL}^{s}\text{–}\mathrm{Cact} \right]$ | Eq. 9 |
| $0={d\left[ \mathrm{DL}^{u}.\mathrm{DL}_{n}^{u} \right]}/{dt}=-k_{D-}^{u}\left[ \mathrm{DL}^{u}.\mathrm{DL}_{n}^{u} \right]+k_{D+}^{u}\left[ \mathrm{DL}_{n}^{u} \right]^{2}-k_{p+}^{u}\left[ \mathrm{DL}^{u}.\mathrm{DL}_{n}^{u} \right]\left[ P \right]$ $+k_{p-}^{u}\left[ \mathrm{DL}^{u}.\mathrm{DL}^{u}\text{–}P \right]-k_{t-}^{u}\left[ \mathrm{DL}^{u}.\mathrm{DL}_{n}^{u} \right]+k_{t+}^{u}\left[ \mathrm{DL}^{u}.\mathrm{DL}_{c}^{u} \right]$ | Eq. 10 |
| $0={d\left[ \mathrm{DL}^{s}.\mathrm{DL}_{n}^{s} \right]}/{dt}=-k_{D-}^{s}\left[ \mathrm{DL}^{s}.\mathrm{DL}_{n}^{s} \right]+k_{D+}^{s}\left[ \mathrm{DL}_{n}^{s} \right]^{2}-k_{p+}^{s}\left[ \mathrm{DL}^{s}.\mathrm{DL}_{n}^{s} \right]\left[ P \right]$ $+k_{p-}^{s}\left[ \mathrm{DL}^{s}.\mathrm{DL}^{s}\text{–}P \right]-k_{t-}^{s}\left[ \mathrm{DL}^{s}.\mathrm{DL}_{n}^{s} \right]+k_{t+}^{s}\left[ \mathrm{DL}^{s}.\mathrm{DL}_{c}^{s} \right]$ | Eq. 11 |
| $0={d\left[ \mathrm{DL}^{u}.\mathrm{DL}_{n}^{s} \right]}/{dt}=-k_{D-}^{us}\left[ \mathrm{DL}^{u}.\mathrm{DL}_{n}^{s} \right]+k_{D+}^{us}\left[ \mathrm{DL}_{n}^{u} \right]\left[ \mathrm{DL}_{n}^{s} \right]-k_{p+}^{us}\left[ \mathrm{DL}^{u}.\mathrm{DL}_{n}^{s} \right]\left[ P \right]$ $+k_{p-}^{us}\left[ \mathrm{DL}^{u}.\mathrm{DL}^{s}\text{–}P \right]-k_{t-}^{us}\left[ \mathrm{DL}^{u}.\mathrm{DL}_{n}^{s} \right]+k_{t+}^{us}\left[ \mathrm{DL}^{u}.\mathrm{DL}_{c}^{s} \right]$ | Eq. 12 |
| $0={d\left[ \mathrm{DL}_{n}^{u} \right]}/{dt}=-2k_{D+}^{u}\left[ \mathrm{DL}_{n}^{u} \right]^{2}+2k_{D-}^{u}\left[ \mathrm{DL}^{u}.\mathrm{DL}_{n}^{u} \right]-k_{D+}^{us}\left[ \mathrm{DL}_{n}^{u} \right]\left[ \mathrm{DL}_{n}^{s} \right]+k_{D-}^{us}\left[ \mathrm{DL}^{u}.\mathrm{DL}_{n}^{s} \right]$ | Eq. 13 |
| $0={d\left[ \mathrm{DL}_{n}^{s} \right]}/{dt}=-2k_{D+}^{s}\left[ \mathrm{DL}_{n}^{s} \right]^{2}+2k_{D-}^{s}\left[ \mathrm{DL}^{s}.\mathrm{DL}_{n}^{s} \right]-k_{D+}^{us}\left[ \mathrm{DL}_{n}^{u} \right]\left[ \mathrm{DL}_{n}^{s} \right]+k_{D-}^{us}\left[ \mathrm{DL}^{u}.\mathrm{DL}_{n}^{s} \right]$ | Eq. 14 |
| $0={d\left[ \mathrm{DL}^{u}.\mathrm{DL}^{u}\text{–}P \right]}/{dt}=k_{p+}^{u}\left[ \mathrm{DL}^{u}.\mathrm{DL}_{n}^{u} \right]\left[ P \right]-k_{p-}^{u}\left[ \mathrm{DL}^{u}.\mathrm{DL}^{u}\text{–}P \right]$ | Eq. 15 |
| $0={d\left[ \mathrm{DL}^{s}.\mathrm{DL}^{s}\text{–}P \right]}/{dt}=k_{p+}^{s}\left[ \mathrm{DL}^{s}.\mathrm{DL}_{n}^{s} \right]\left[ P \right]-k_{p-}^{s}\left[ \mathrm{DL}^{s}.\mathrm{DL}^{s}\text{–}P \right]$ | Eq. 16 |
| $0={d\left[ \mathrm{DL}^{u}.\mathrm{DL}^{s}\text{–}P \right]}/{dt}=k_{p+}^{us}\left[ \mathrm{DL}^{u}.\mathrm{DL}_{n}^{s} \right]\left[ P \right]-k_{p-}^{us}\left[ \mathrm{DL}^{u}.\mathrm{DL}^{s}\text{–}P \right]$ | Eq. 17 |
| $0={d\left[ P \right]}/{dt}=-k_{p+}^{us}\left[ \mathrm{DL}^{u}.\mathrm{DL}_{n}^{s} \right]\left[ P \right]+k_{p-}^{us}\left[ \mathrm{DL}^{u}.\mathrm{DL}^{s}\text{–}P \right]-k_{p+}^{u}\left[ \mathrm{DL}^{u}.\mathrm{DL}_{n}^{u} \right]\left[ P \right]$ $+k_{p-}^{u}\left[ \mathrm{DL}^{u}.\mathrm{DL}^{u}\text{–}P \right]-k_{p+}^{s}\left[ \mathrm{DL}^{s}.\mathrm{DL}_{n}^{s} \right]\left[ P \right]+k_{p-}^{s}\left[ \mathrm{DL}^{s}.\mathrm{DL}^{s}\text{–}P \right]$ | Eq. 18 |
| ${DL}_{Total}^{u}=\left[ \mathrm{DL}_{c}^{u} \right]+\left[ \mathrm{DL}_{n}^{u} \right]+\left[ \mathrm{DL}^{u}.\mathrm{DL}_{c}^{s} \right]+\left[ \mathrm{DL}^{u}.\mathrm{DL}_{n}^{s} \right]+\left[ \mathrm{DL}^{u}.\mathrm{DL}^{s}\text{–}\mathrm{Cact} \right]+\left[ \mathrm{DL}^{u}.\mathrm{DL}^{s}\text{–}P \right]$ $+2 \left[ \mathrm{DL}^{u}.\mathrm{DL}_{c}^{u} \right]+2 \left[ \mathrm{DL}^{u}.\mathrm{DL}_{n}^{u} \right]+2 \left[ \mathrm{DL}^{u}.\mathrm{DL}^{u}\text{–}\mathrm{Cact} \right]+2 \left[ \mathrm{DL}^{u}.\mathrm{DL}^{u}\text{–}P \right]$ | Eq. 19 |
| ${DL}_{Total}^{s}=\left[ \mathrm{DL}_{c}^{s} \right]+\left[ \mathrm{DL}_{n}^{s} \right]+\left[ \mathrm{DL}^{u}.\mathrm{DL}_{c}^{s} \right]+\left[ \mathrm{DL}^{u}.\mathrm{DL}_{n}^{s} \right]+\left[ \mathrm{DL}^{u}.\mathrm{DL}^{s}\text{–}\mathrm{Cact} \right]+\left[ \mathrm{DL}^{u}.\mathrm{DL}^{s}\text{–}P \right]$ $+2 \left[ \mathrm{DL}^{s}.\mathrm{DL}_{c}^{s} \right]+2 \left[ \mathrm{DL}^{s}.\mathrm{DL}_{n}^{s} \right]+2 \left[ \mathrm{DL}^{s}.\mathrm{DL}^{s}\text{–}\mathrm{Cact} \right]+2 \left[ \mathrm{DL}^{s}.\mathrm{DL}^{s}\text{–}P \right]$ | Eq. 20 |
| $P_{Total}=\left[ P \right]+\left[ \mathrm{DL}^{u}.\mathrm{DL}^{u}\text{–}P \right]+\left[ \mathrm{DL}^{s}.\mathrm{DL}^{s}\text{–}P \right]+\left[ \mathrm{DL}^{u}.\mathrm{DL}^{s}\text{–}P \right]$ | Eq. 21 |
| ${Cact}_{Total}=\left[ \mathrm{Cact} \right]+\left[ \mathrm{DL}^{u}.\mathrm{DL}^{u}\text{–}\mathrm{Cact} \right]+\left[ \mathrm{DL}^{s}.\mathrm{DL}^{s}\text{–}\mathrm{Cact} \right]+\left[ \mathrm{DL}^{u}.\mathrm{DL}^{s}\text{–}\mathrm{Cact} \right]$ | Eq. 22 |

The algebraic expressions in combination with conservation of total DL, binding sites and Cact are used to solve for $\left[ \mathrm{DL}_{c}^{u} \right]$, $\left[ \mathrm{DL}_{c}^{s} \right]$ as functions of total DL concentrations. This gives the following two equations (Eq. 23-24) for the wild-type.

| ${DL}_{Total}^{u}=\left[ \mathrm{DL}_{c}^{u} \right]\times\left( 1+\sqrt{K_{t}^{u}}+K_{D}^{us}\left( 1+K_{t}^{us} \right) \left[ \mathrm{DL}_{c}^{s} \right]+{2K}_{D}^{u}\left( 1+K_{t}^{u} \right) \left[ \mathrm{DL}_{c}^{u} \right] \right)$ $+\frac{\left[ \mathrm{DL}_{c}^{u} \right] {Cact}_{Total} \left( {{2K}_{D}^{u}K}_{i}^{u} \left[ \mathrm{DL}_{c}^{u} \right]+{K_{D}^{us}K}_{i}^{us} \left[ \mathrm{DL}_{c}^{s} \right] \right)}{1+K_{D}^{u}K_{i}^{u} \left[ \mathrm{DL}_{c}^{u} \right]^{2}+K_{D}^{s}K_{i}^{s} \left[ \mathrm{DL}_{c}^{s} \right]^{2}+K_{D}^{us}K_{i}^{us} \left[ \mathrm{DL}_{c}^{u} \right] \left[ \mathrm{DL}_{c}^{s} \right]}$ $+\frac{\left[ \mathrm{DL}_{c}^{u} \right] P_{Total} \left( {{{2K}_{D}^{u}K}_{p}^{u}K}_{t}^{u} \left[ \mathrm{DL}_{c}^{u} \right]+{{K_{D}^{us}K}_{p}^{us}K}_{t}^{us} \left[ \mathrm{DL}_{c}^{s} \right] \right)}{1+K_{D}^{u}K_{p}^{u}K_{t}^{u} \left[ \mathrm{DL}_{c}^{u} \right]^{2}+K_{D}^{s}K_{p}^{s}K_{t}^{s} \left[ \mathrm{DL}_{c}^{s} \right]^{2}+K_{D}^{us}K_{p}^{us}K_{t}^{us} \left[ \mathrm{DL}_{c}^{u} \right] \left[ \mathrm{DL}_{c}^{s} \right]}$ | Eq. 23 |
| --- | --- |
| ${DL}_{Total}^{s}=\left[ \mathrm{DL}_{c}^{s} \right]\times\left( 1+\sqrt{K_{t}^{s}}+K_{D}^{us}\left( 1+K_{t}^{us} \right) \left[ \mathrm{DL}_{c}^{u} \right]+{2K}_{D}^{s}\left( 1+K_{t}^{s} \right) \left[ \mathrm{DL}_{c}^{s} \right] \right)$ $+\frac{\left[ \mathrm{DL}_{c}^{s} \right] {Cact}_{Total} \left( {{2K}_{D}^{s}K}_{i}^{s} \left[ \mathrm{DL}_{c}^{s} \right]+{K_{D}^{us}K}_{i}^{us} \left[ \mathrm{DL}_{c}^{u} \right] \right)}{1+K_{D}^{u}K_{i}^{u} \left[ \mathrm{DL}_{c}^{u} \right]^{2}+K_{D}^{s}K_{i}^{s} \left[ \mathrm{DL}_{c}^{s} \right]^{2}+K_{D}^{us}K_{i}^{us} \left[ \mathrm{DL}_{c}^{u} \right] \left[ \mathrm{DL}_{c}^{s} \right]}$ $+\frac{\left[ \mathrm{DL}_{c}^{s} \right] P_{Total} \left( {{{2K}_{D}^{s}K}_{p}^{s}K}_{t}^{s} \left[ \mathrm{DL}_{c}^{s} \right]+{{K_{D}^{us}K}_{p}^{us}K}_{t}^{us} \left[ \mathrm{DL}_{c}^{u} \right] \right)}{1+K_{D}^{u}K_{p}^{u}K_{t}^{u} \left[ \mathrm{DL}_{c}^{u} \right]^{2}+K_{D}^{s}K_{p}^{s}K_{t}^{s} \left[ \mathrm{DL}_{c}^{s} \right]^{2}+K_{D}^{us}K_{p}^{us}K_{t}^{us} \left[ \mathrm{DL}_{c}^{u} \right] \left[ \mathrm{DL}_{c}^{s} \right]}$ | Eq. 24 |

For simplicity, SUMOylated heterodimer $\mathrm{DL}^{u}.\mathrm{DL}^{s}$ was assumed to have the same properties and affinities as the SUMOylated homodimer $\mathrm{DL}^{s}.\mathrm{DL}^{s}$ (i.e., $K_{D}^{us}=K_{D}^{s}, K_{i}^{us}=K_{i}^{s}, K_{p}^{us}=K_{p}^{s},$ and $k^{us}=k^{s}$). Furthermore, the pseudo steady state assumption yields an additional constraint on the transport of SUMOylated heterodimer, given by

| $K_{t}^{us}=\sqrt{K_{t}^{u}\times K_{t}^{s}}$ | Eq. 25 |
| --- | --- |

Equations 23 and 24 are numerically solved using reported parameter values (SI Table 1) and the rate of reporter expression is calculated using

| $R^{WT}=\frac{d\left[ \mathrm{mRNA} \right]}{dt}=k^{u} \left[ \mathrm{DL}^{u}.\mathrm{DL}^{u}\text{–}P \right]+k^{s} \left[ \mathrm{DL}^{s}.\mathrm{DL}^{s}\text{–}P \right]+k^{s} \left[ \mathrm{DL}^{u}.\mathrm{DL}^{s}\text{–}P \right]$ | Eq. 26 |
| --- | --- |

For WT, the total DL reported by Tay et al. is assumed to comprise of 95% DL^U^ and 5% DL^S^; whereas for DL^SCR^, the entire DL is assumed to be unSUMOylated (${DL}_{Total}^{s}=0$). That is, for the SCR mutant,

| ${DL}_{Total}^{u}=\left[ \mathrm{DL}_{c}^{u} \right]\times\left( 1+\sqrt{K_{t}^{u}}+2K_{D}^{u}\left( 1+K_{t}^{u} \right) \left[ \mathrm{DL}_{c}^{u} \right] \right)+\frac{2K_{D}^{u}K_{i}^{u} Cact_{Total} \left[ \mathrm{DL}_{c}^{u} \right]^{2}}{1+K_{D}^{u}K_{i}^{u} \left[ \mathrm{DL}_{c}^{u} \right]^{2}}+\frac{2K_{D}^{u}K_{p}^{u}K_{t}^{u} P_{Total} \left[ \mathrm{DL}_{c}^{u} \right]^{2}}{1+K_{D}^{u}K_{p}^{u}K_{t}^{u} \left[ \mathrm{DL}_{c}^{u} \right]^{2}}$ | Eq. 27 |
| --- | --- |
| $R^{SCR}=\frac{d\left[ \mathrm{mRNA} \right]}{dt}=k^{u} \left[ \mathrm{DL}^{u}.\mathrm{DL}^{u}\text{–}P \right]$ | Eq. 28 |

Similar to WT, Eq. 27 is also numerically solved using the *fsolve* function in MATLAB, with the rate of reporter gene expression having contributions only from unSUMOylated DL. We assume that the effect of SUMOylation is to change the values of the specific reaction rate constants for reactions involving DL. For instance, the specific transcription rate ($k^{s}$) when promoter is bound to $\mathrm{DL}^{s}.\mathrm{DL}^{s}$ or $\mathrm{DL}^{u}.\mathrm{DL}^{s}$ might be higher or lower than the rate ($k^{u}$) when promoter is bound to $\mathrm{DL}^{u}.\mathrm{DL}^{u}$. The relative reporter expression (${R^{SCR}}/{R^{WT}}$) is calculated at different values of the relative reaction rate constants for SUMOylated and unSUMOylated variants by solving equations with rate constants set at each of those values.
