# Supplementary material for: SUMOylation of Dorsal attenuates Toll/NF-κB signaling
Source: Genetics. 2022 May 14;221(3):iyac081. doi: 10.1093/genetics/iyac081 (PMC9252280; doi:10.1093/genetics/iyac081)
Supplement: iyac081_Supplemental_Material_Legends [file iyac081_supplemental_material_legends.docx]

**Supplementary Figures**

Fig. S1. *dl* transcripts express at similar levels in *dl^WT^* and *dl^SCR^.*

Fig. S2. Haplo-sufficiency of the *dl^SCR^* allele.

Fig. S3. The DL gradient visualized with a DL antibody in WT, *dl^WT^/Df*, and *dl^SCR^/Df* embryos.

Fig. S4. Quantitative changes in the transcriptome of embryos laid by *dl^SCR^* mothers.

Fig. S5. *dl^1^* is a S317N mutant and is resistant to Toll signaling.

Fig. S6. Crystal cells in *dl^SCR^*, *dl^1^/dl^SCR^*, and *dl^4^/dl^SCR^* larvae.

Fig. S7. Simulating the effect of DL SUMOylation.

Fig. S8. Summary of known physical interactors of DL.

Fig. S9. Simulating the effect of a lower fraction of SUMOylated DL.

Fig. S10. Simulating the effect of a higher fraction of SUMOylated DL.

Fig. S11. Simulating the effect of increase in cytosolic Cact levels.

Fig. S12. Simulating the effect of 10X lower cytosolic Cact levels.

Fig. S13. Simulating the effect of 100X lower cytosolic Cact levels.

**Supplementary Text**

SI-1. Mathematical model.

SI-2. Parameter table.

**Supplementary Tables**

ST-1. Differentially expressed genes in the 0-2 hour embryo across all four genotypes studied, *dl^WT^*, *dl^SCR^*, *dl^WT^*/*Df,* *dl^SCR^*/*Df*.
